# Supplementary material for: Tracing animal genomic evolution with the chromosomal-level assembly of the freshwater sponge Ephydatia muelleri
Source: Nat Commun. 2020 Jul 27;11:3676. doi: 10.1038/s41467-020-17397-w (PMC7385117; doi:10.1038/s41467-020-17397-w)
Supplement: Supplementary file 3 — Reporting Summary [file 41467_2020_17397_MOESM3_ESM.pdf]

nature research

Corresponding author(s): Sally Leys and Nathan Kenny

Last updated by author(s): Jun 16, 2020

Reporting Summary

Nature Research wishes to improve the reproducibility of the work that we publish. This form provides structure for consistency and transparency in reporting. For further information on Nature Research policies, see Authors & Referees and the Editorial Policy Checklist .

Statistics

For all statistical analyses, confirm that the following items are present in the figure legend, table legend, main text, or Methods section.

n/a	Confirmed	
☐	☒	The exact sample size (*n*) for each experimental group/condition, given as a discrete number and unit of measurement
☐	☒	A statement on whether measurements were taken from distinct samples or whether the same sample was measured repeatedly
☐	☒	The statistical test(s) used AND whether they are one- or two-sided<br>*Only common tests should be described solely by name; describe more complex techniques in the Methods section.*
☒	☐	A description of all covariates tested
☐	☒	A description of any assumptions or corrections, such as tests of normality and adjustment for multiple comparisons
☒	☐	A full description of the statistical parameters including central tendency (e.g. means) or other basic estimates (e.g. regression coefficient) AND variation (e.g. standard deviation) or associated estimates of uncertainty (e.g. confidence intervals)
☐	☒	For null hypothesis testing, the test statistic (e.g. *F*, *t*, *r*) with confidence intervals, effect sizes, degrees of freedom and *P* value noted<br>*Give P values as exact values whenever suitable.*
☐	☒	For Bayesian analysis, information on the choice of priors and Markov chain Monte Carlo settings
☒	☐	For hierarchical and complex designs, identification of the appropriate level for tests and full reporting of outcomes
☒	☐	Estimates of effect sizes (e.g. Cohen's *d*, Pearson's *r*), indicating how they were calculated

Our web collection on statistics for biologists contains articles on many of the points above.

Software and code

Policy information about availability of computer code

Data collection

The genome was assembled using the FALCON 1.8.8 pipeline from Pacific Biosciences, polished through PacBio's Arrow algorithm from SMRT Link 5.0.1. Shotgun and Chicago library sequences were aligned to the draft input assembly using a modified SNAP read mapper (http://snap.cs.berkeley.edu). The separations of Chicago read pairs mapped within draft scaffolds were analyzed by HiRiSE (Dovetail Genomics). After aligning and scaffolding Chicago data, Dovetail HiC library sequences were aligned and scaffolded following the same method.

Data analysis

Custom code used to analyse data in this study are available as Supplementary Files and have also been uploaded to https://
spaces.facsci.ualberta.
ca/ephybase/ and https://bitbucket.org/EphydatiaGenome/.

Genome annotation
Gene models were predicted using AUGUSTUS3.3.2 annotation software (http//bioinf.uni-greifswald.de/augustus/) with previously published RNAseq datasets used for training. As the basis for gene prediction, the non-masked genome was used, to avoid artefacts, missed exons or missing gene portions caused by masked areas of the genome. The BUSCO v2/3 set was used in genome mode to determine gene recovery metrics. RepeatModeler 2.0 and RepeatMasker 4.1.0 were used sequentially to predict repetitive content within the genome. Contamination and bacterial content was excluded by BLAST 2.10.0 against a range of well-annotated databases. Syntenic relationships were assessed using reciprocal blasts and custom python scripts (scaffold_synteny.py, see bitbucket repository https://bitbucket.org/E.Genome/ or Supplementary File 2). Taxonomy assessment of identified symbiont sequence was performed in CheckM 1.10.0, MiGA and other software, as detailed in Supplementary File 1 S4. Automated annotation of gene sequences was performed using DIAMOND 0.9.31 BLASTx against the nr and swissprot databases followed by functional annotation with BLAST2GO PRO. Methylation studies were performed using the MethylC-seq protocol. TADs and loops were identified using HOMER v4.11. Orthogroup based analyses were performed primarily using Orthofinder2, with IQtree v1.6.12, MAFFT 7.450 and diamond BLAST options (described in detail Supplementary File 1, S7). Selection tests were performed according to the methodology put forward by Santagata (https://github.com/Santagata/Select_Test).

Developmental gene expression: RNAseq was performed by LC Sciences (Houston, Tx). HISAT 2.0 was used to map RNAseq reads to the reference E. muelleri genome. edgeR 3.30.3 was used to estimate the expression levels of all transcripts across all replicate samples.
Gene family content was assessed using targeted manual BLAST 2.10.0, with HMMER v3.3 used when necessary to test absence. Reciprocal BLAST was used to ensure assignment of identity, with the identity of key gene families assessed using phylogenetic inference as shown in Supplementary File 1. Holobiont content from a number of E. muelleri samples was assessed with Mothur v.1.41.3 and an adaptation of MiSeq SOP protocol.

For manuscripts utilizing custom algorithms or software that are central to the research but not yet described in published literature, software must be made available to editors/reviewers. We strongly encourage code deposition in a community repository (e.g. GitHub). See the Nature Research guidelines for submitting code & software for further information.

Data

Policy information about availability of data

All manuscripts must include a data availability statement. This statement should provide the following information, where applicable:

- Accession codes, unique identifiers, or web links for publicly available datasets
- A list of figures that have associated raw data
- A description of any restrictions on data availability

A browsable version of the genome of Ephydatia muelleri, gene predictions, a masked version of the assembly, and a variety of annotation formats are available from https://spaces.facsci.ualberta.ca/ephybase/. This project has been deposited at DDBJ/ENA/GenBank under the accession JABACO000000000. The version described in this paper is version JABACO010000000. The sequence of Flavobacterium sp. has also been uploaded, with accession number CP051546. The raw reads have been uploaded to the NCBI SRA at accession number PRJNA579531 / GEO GSE139500.

Field-specific reporting

Please select the one below that is the best fit for your research. If you are not sure, read the appropriate sections before making your selection.

- [x] Life sciences
- [ ] Behavioural & social sciences
- [ ] Ecological, evolutionary & environmental sciences

For a reference copy of the document with all sections, see nature.com/documents/nr-reporting-summary-flat.pdf

Life sciences study design

All studies must disclose on these points even when the disclosure is negative.

Sample size	For genome sequencing, a single individual was used. This is the correct number to use to avoid heterozygosity and the golden standard in genome projects.
Data exclusions	No data exclusions are relevant
Replication	Replication was primarily relevant for the RNAseq component of this study. We used 3 replicate samples per timepoint to ensure the consistency of our results and replication was successful.
Randomization	Randomization is not relevant to this, largely descriptive, study
Blinding	Blinding is not relevant to this study in any way.

Reporting for specific materials, systems and methods

We require information from authors about some types of materials, experimental systems and methods used in many studies. Here, indicate whether each material, system or method listed is relevant to your study. If you are not sure if a list item applies to your research, read the appropriate section before selecting a response.

Materials & experimental systems

n/a	Involved in the study	
x		Antibodies
x		Eukaryotic cell lines
x		Palaeontology
	x	Animals and other organisms
x		Human research participants
x		Clinical data

Methods

n/a	Involved in the study	
x		ChIP-seq
x		Flow cytometry
x		MRI-based neuroimaging

Animals and other organisms

Policy information about studies involving animals; ARRIVE guidelines recommended for reporting animal research

Laboratory animals	The study did not involve laboratory animals
Wild animals	The study did not involve wild animals, except the individuals transported to the laboratory (see Field-collected samples below)
Field-collected samples	For DNA sequencing a single clone was collected as an overwintering cysts (gemmule) from the Sooke Reservoir, at the head tank of the city of Victoria, British Columbia drinking water system. A voucher specimen is deposited with the Royal British Columbia Museum (RBCM019-00140-001).<br>For transcriptomic sequencing, gemmules from three individuals stored in 10% DMSO at -80ºC for 1-5 years were thawed and hatched. For stage 1, gemmules were allowed to develop for only 12 hours before tissue was harvested for RNA. For stages 2, 3, and 5, gemmules were allowed to hatch and sponges cultured in the lab as described previously (Leys et al. 2019). Tissue was flash frozen in liquid nitrogen and either stored at -80ºC or processed immediately for RNA.<br>For amplicon sequencing we collected gemmules and tissue containing gemmules from adult sponges in 6 locations in the northern hemisphere (Supplementary Table 21, Supplementary Figure 37A). Both unhatched and hatched gemmules were analysed, as well as adult tissues containing gemmules. Gemmules from the adult tissue were removed as much as possible before DNA extraction. When gemmules were hatched, hatching was performed for 1 week following previously published protocols (Leys et al. 2019). Each sample was amplified and sequenced in duplicate (pseudoreplicates a and b).<br><br>Leys, S., Grombacher, L. & Hill, A. Hatching and freezing gemmules from the freshwater sponge Ephydatia muelleri v1 (protocols.io.863hzgn). protocols.io (2019). doi:10.17504/protocols.io.863hzgn
Ethics oversight	No ethical approval is relevant or required for work with sponges in any country

Note that full information on the approval of the study protocol must also be provided in the manuscript.
