# Supplementary material for: Tracing animal genomic evolution with the chromosomal-level assembly of the freshwater sponge Ephydatia muelleri
Source: Nat Commun. 2020 Jul 27;11:3676. doi: 10.1038/s41467-020-17397-w (PMC7385117; doi:10.1038/s41467-020-17397-w)
Supplement: Supplementary file 4 — Description of Additional Supplementary Files [file 41467_2020_17397_MOESM4_ESM.pdf]

Description of Additional Supplementary Files

File Name: Supplementary Data 1

Description: *Flavobacterium* sp. sequence, predicted proteins and cds sequences

File Name: Supplementary Data 2

Description: BLAST/functional annotation results, *Ephydatia muelleri* gene annotations

File Name: Supplementary Data 3
Description: Comparative genome statistics and scripts used in generating figures

File Name: Supplementary Data 4
Description: Synteny analysis, additional plots and data

File Name: Supplementary Data 5
Description: Full lists of genes, gain/loss analyses

File Name: Supplementary Data 6
Description: Full results and orthogroups, positive selection analyses

File Name: Supplementary Data 7
Description: Clustering analyses, lists of genes in each cluster

File Name: Supplementary Data 8
Description: SNARE data, full gene sequences and annotations

File Name: Supplementary Data 9
Description: Amplicon sequencing data, raw and intermediate results
