# Supplementary material for: Tracing animal genomic evolution with the chromosomal-level assembly of the freshwater sponge Ephydatia muelleri
Source: Nat Commun. 2020 Jul 27;11:3676. doi: 10.1038/s41467-020-17397-w (PMC7385117; doi:10.1038/s41467-020-17397-w)

# Tethya scaffold sizes

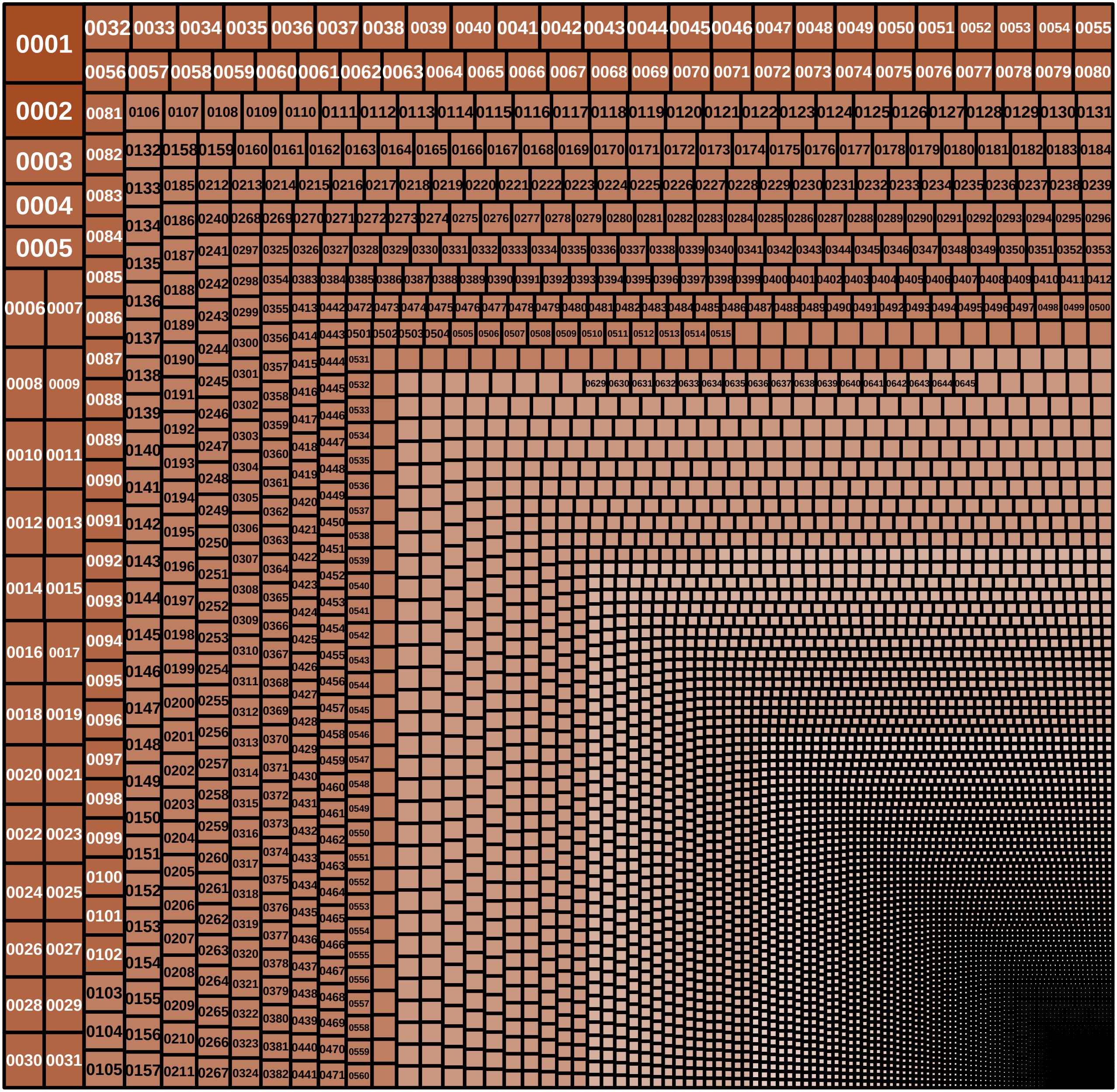

Supplement: Supplementary file 7 — Supplementary Data 3 [file 41467_2020_17397_MOESM7_ESM.zip › Suppl_Data_3_Comp_genome_statistics_scripts/treemap/twi1_treemap.pdf]
