# Supplementary material for: Tracing animal genomic evolution with the chromosomal-level assembly of the freshwater sponge Ephydatia muelleri
Source: Nat Commun. 2020 Jul 27;11:3676. doi: 10.1038/s41467-020-17397-w (PMC7385117; doi:10.1038/s41467-020-17397-w)
Supplement: Supplementary file 7 — Supplementary Data 3 [file 41467_2020_17397_MOESM7_ESM.zip › Suppl_Data_3_Comp_genome_statistics_scripts/treemap/sponge_combined_treemaps_v1.pdf]

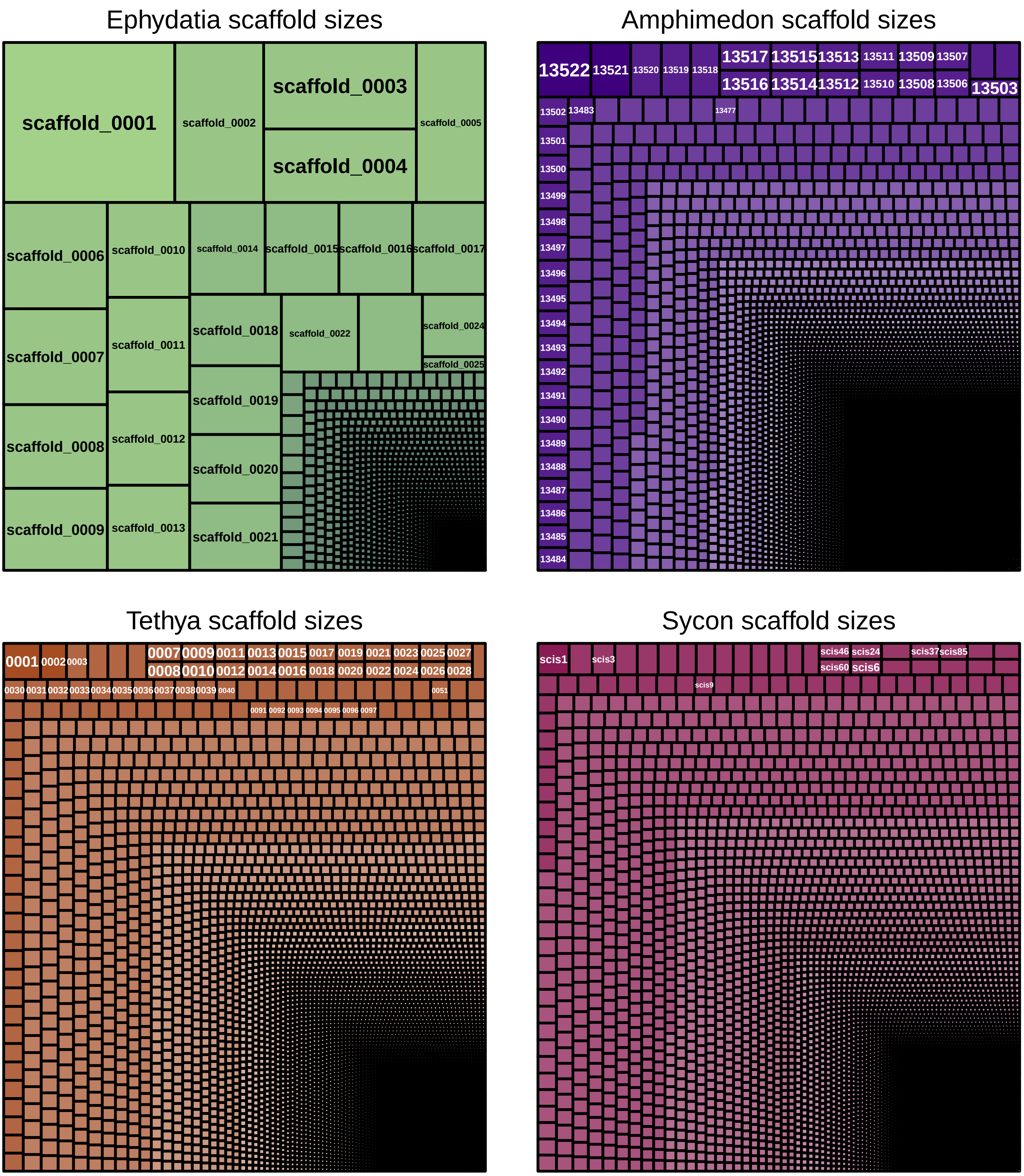
Ephydatia scaffold sizes
scaffold_0001
scaffold_0002
scaffold_0003
scaffold_0004
scaffold_0005
scaffold_0006
scaffold_0010
scaffold_0014
scaffold_0015
scaffold_0016
scaffold_0017
scaffold_0007
scaffold_0011
scaffold_0018
scaffold_0022
scaffold_0024
scaffold_0025
scaffold_0019
scaffold_0008
scaffold_0012
scaffold_0020
scaffold_0009
scaffold_0013
scaffold_0021
Amphimedon scaffold sizes
13522
13521
13520
13519
13518
13517
13515
13513
13511
13509
13507
13516
13514
13512
13510
13508
13506
13503
13502
13483
13477
13501
13500
13499
13498
13497
13496
13495
13494
13493
13492
13491
13490
13489
13488
13487
13486
13485
13484
Tethya scaffold sizes
0001
0002
0003
0007
0009
0011
0013
0015
0017
0019
0021
0023
0025
0027
0008
0010
0012
0014
0016
0018
0020
0022
0024
0026
0028
0030
0031
0032
0033
0034
0035
0036
0037
0038
0039
0040
0051
0091
0092
0093
0094
0095
0096
0097
Sycon scaffold sizes
scis1
scis3
scis46
scis24
scis37
scis85
scis60
scis6
scis9
