# Supplementary figures and images for: Tracing animal genomic evolution with the chromosomal-level assembly of the freshwater sponge Ephydatia muelleri
Source: Nat Commun. 2020 Jul 27;11:3676. doi: 10.1038/s41467-020-17397-w (PMC7385117; doi:10.1038/s41467-020-17397-w)

### Exon lengths

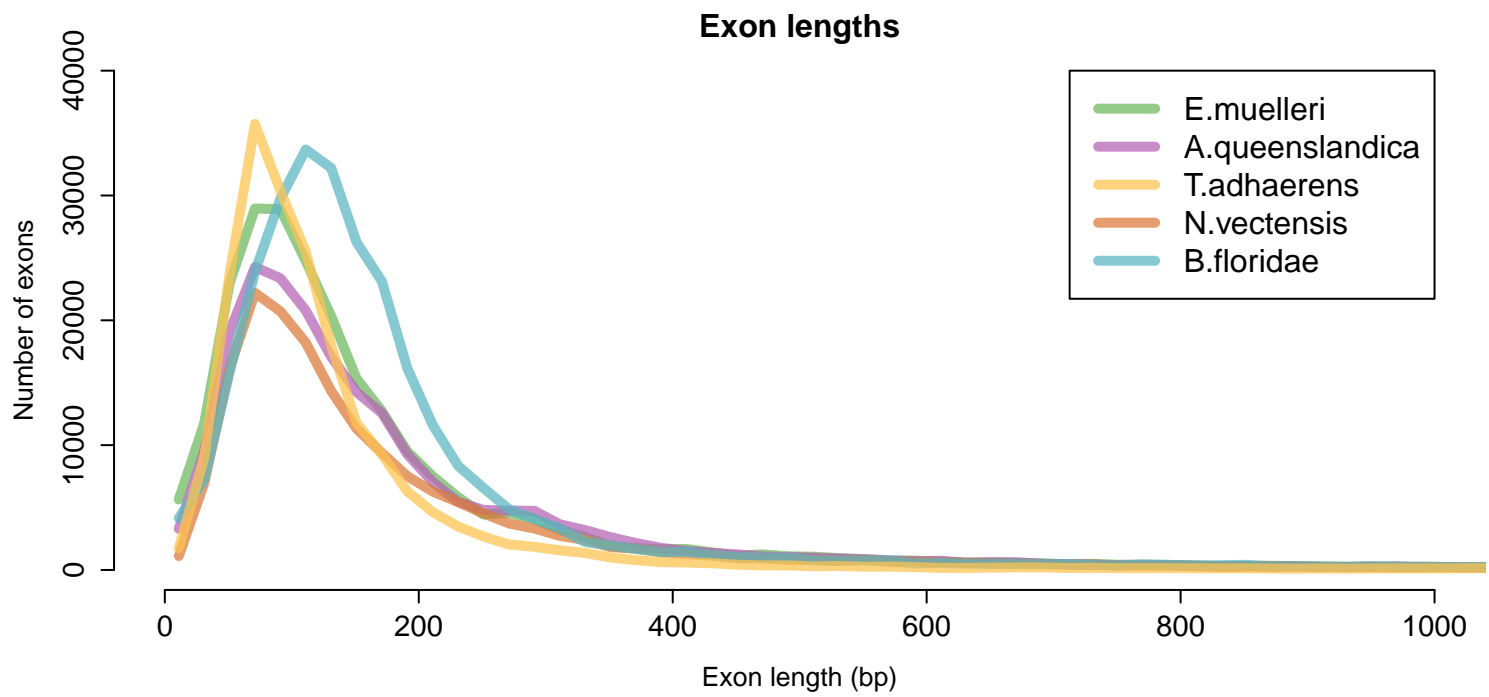

### Intron lengths

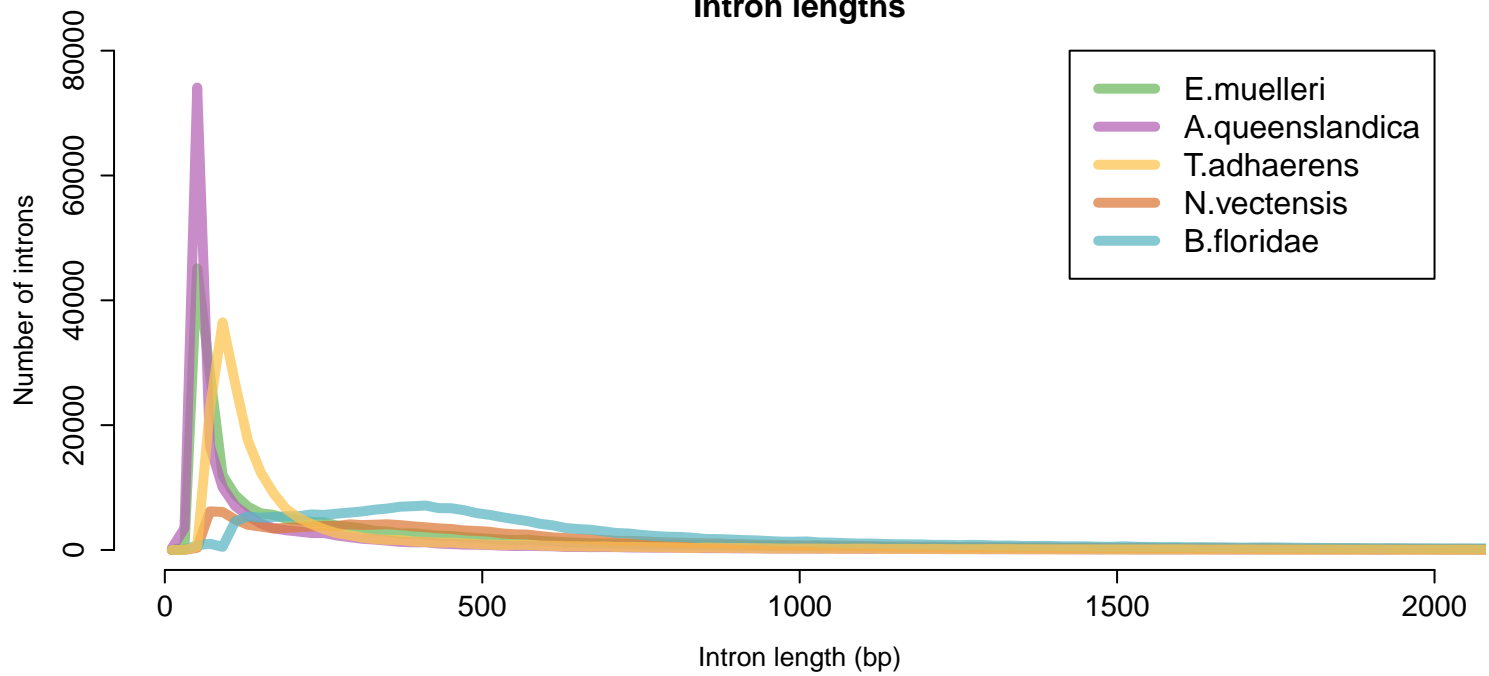

### Intergenic lengths

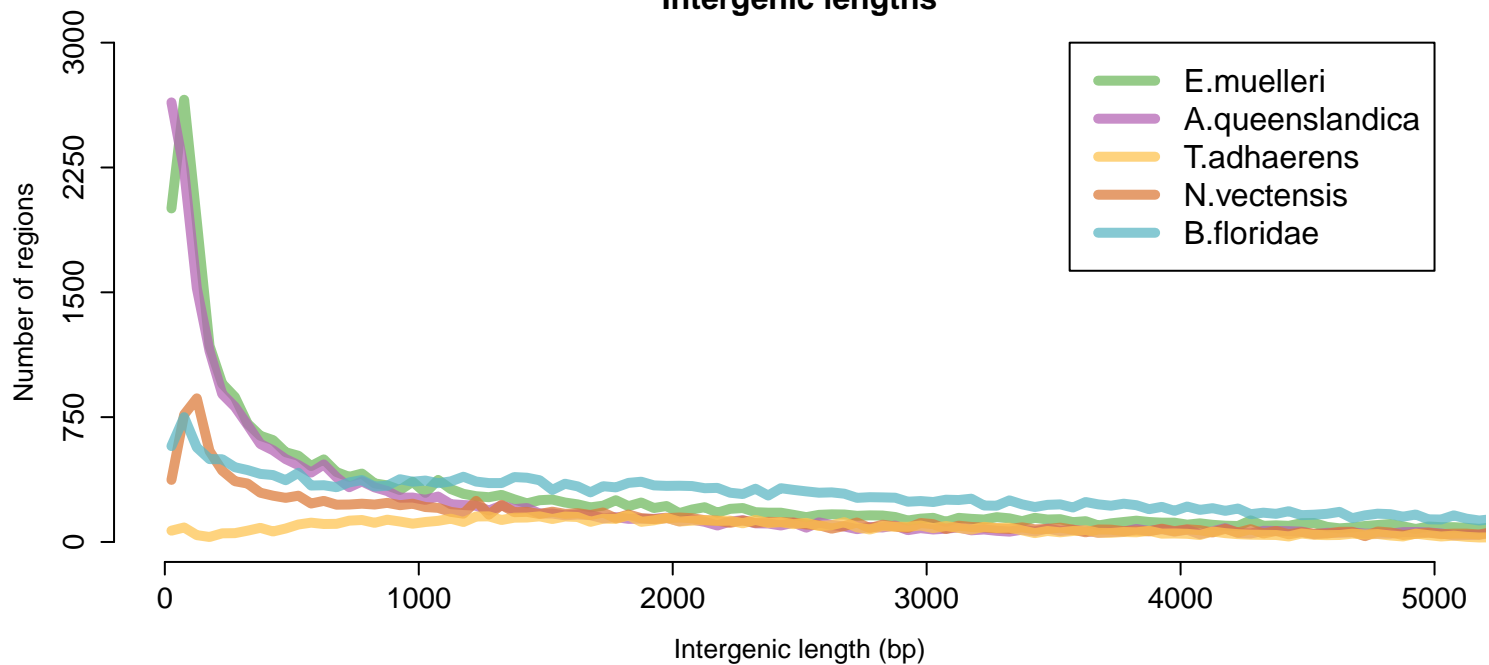

Supplement: Supplementary file 7 — Supplementary Data 3 [file 41467_2020_17397_MOESM7_ESM.zip › Suppl_Data_3_Comp_genome_statistics_scripts/intron-exon/multi_species_exon_intron_hist.pdf]

Sycon scaffold sizes

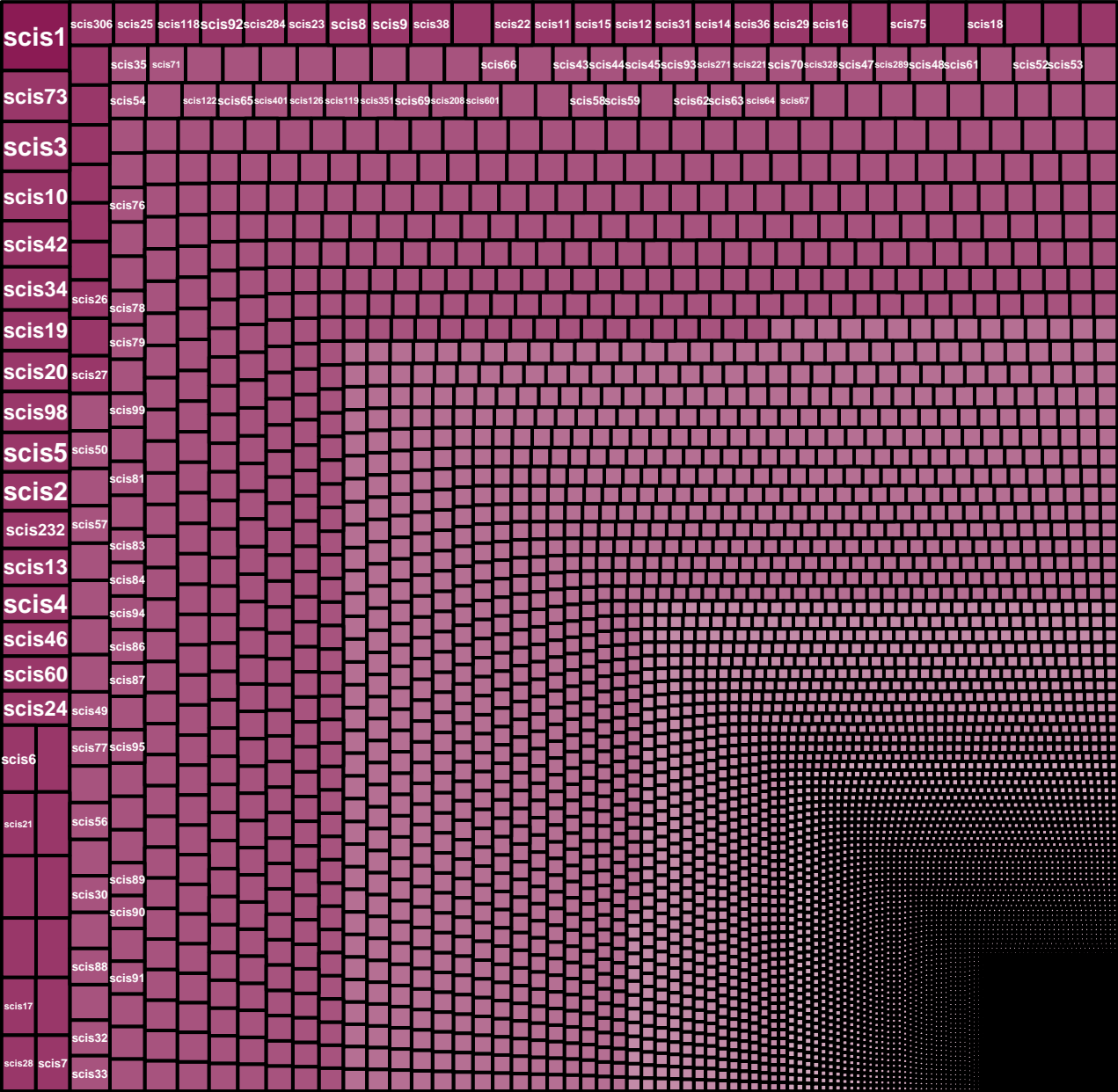

Supplement: Supplementary file 7 — Supplementary Data 3 [file 41467_2020_17397_MOESM7_ESM.zip › Suppl_Data_3_Comp_genome_statistics_scripts/treemap/scil1_treemap.pdf]

322Mb

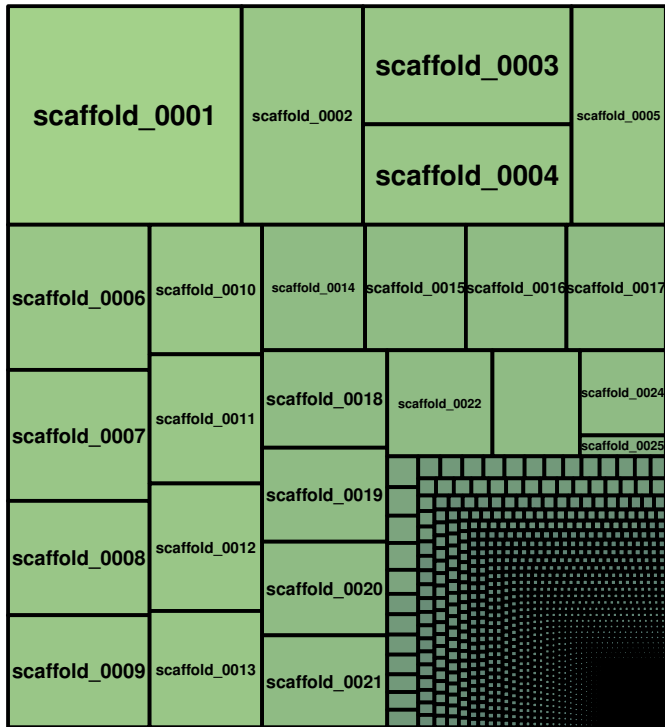

*Amphimedon queenslandica*

166Mb

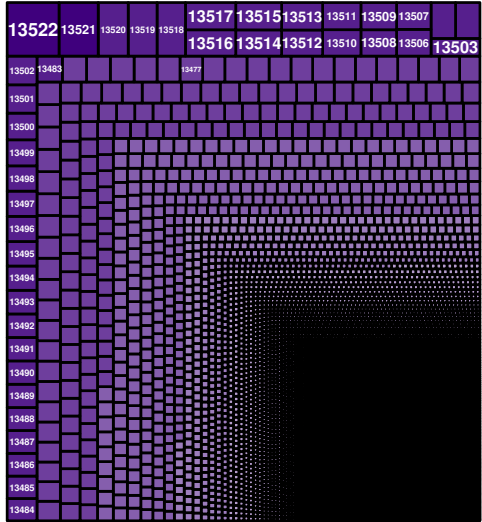

*Tethya wilhelma*

125Mb

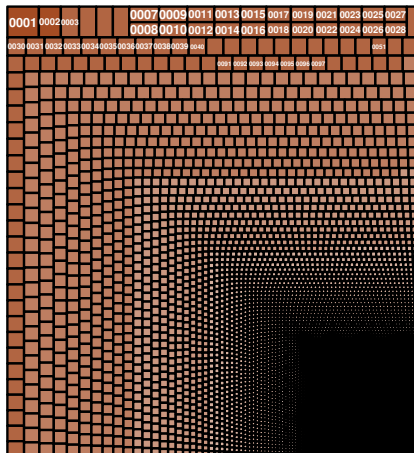

*Sycon ciliatum* 357Mb

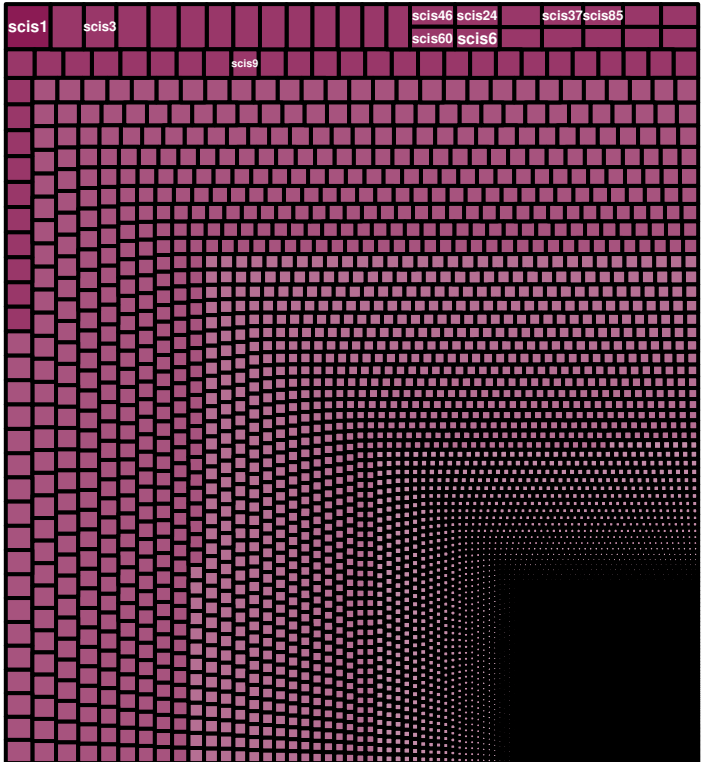

Supplement: Supplementary file 7 — Supplementary Data 3 [file 41467_2020_17397_MOESM7_ESM.zip › Suppl_Data_3_Comp_genome_statistics_scripts/treemap/sponge_combined_treemaps_v2.pdf]

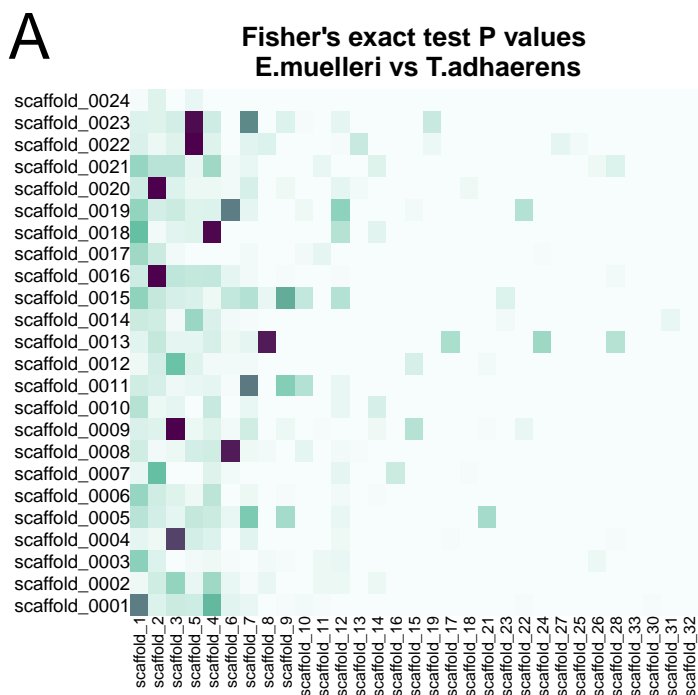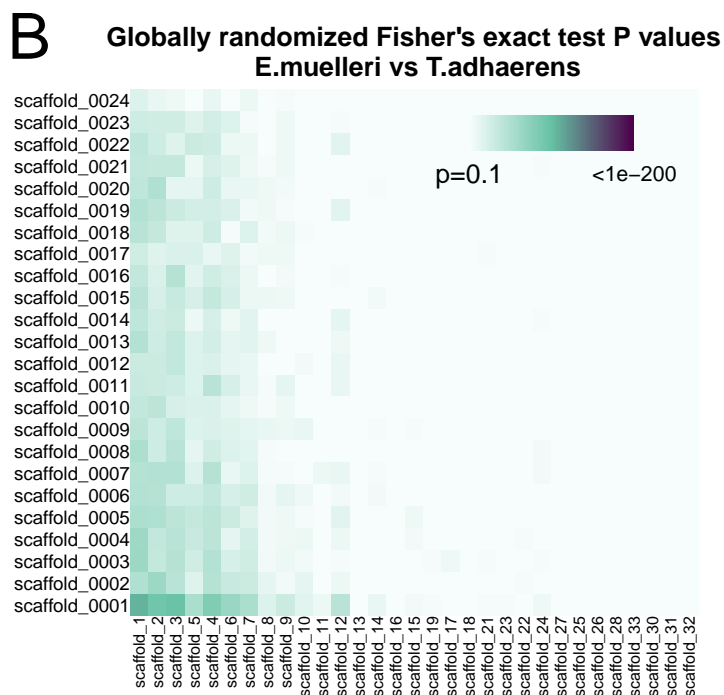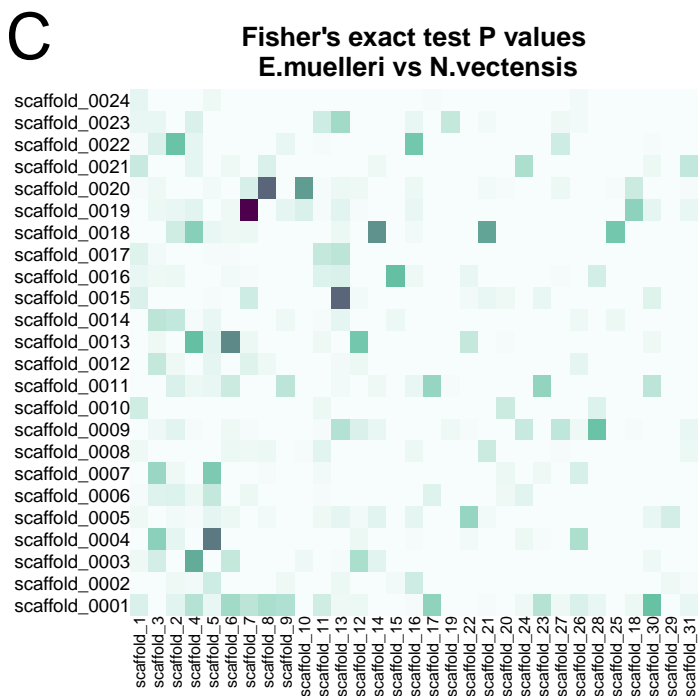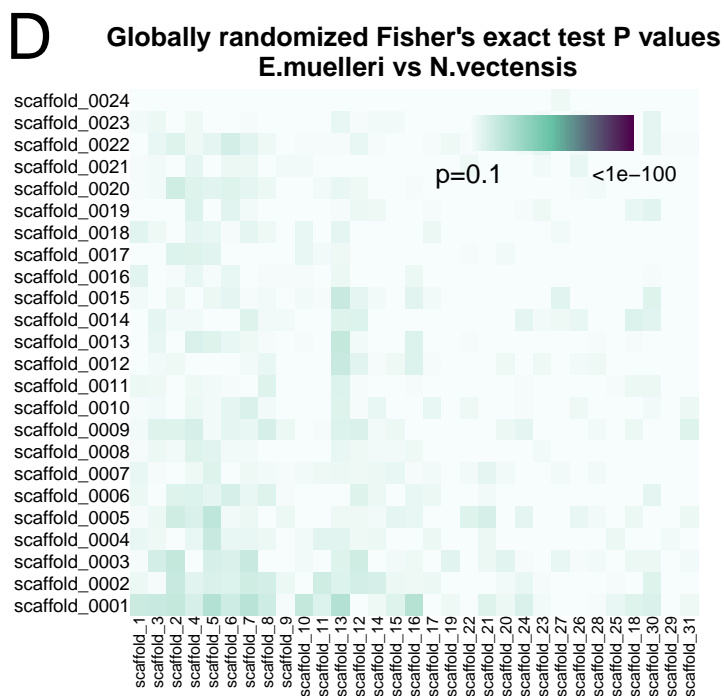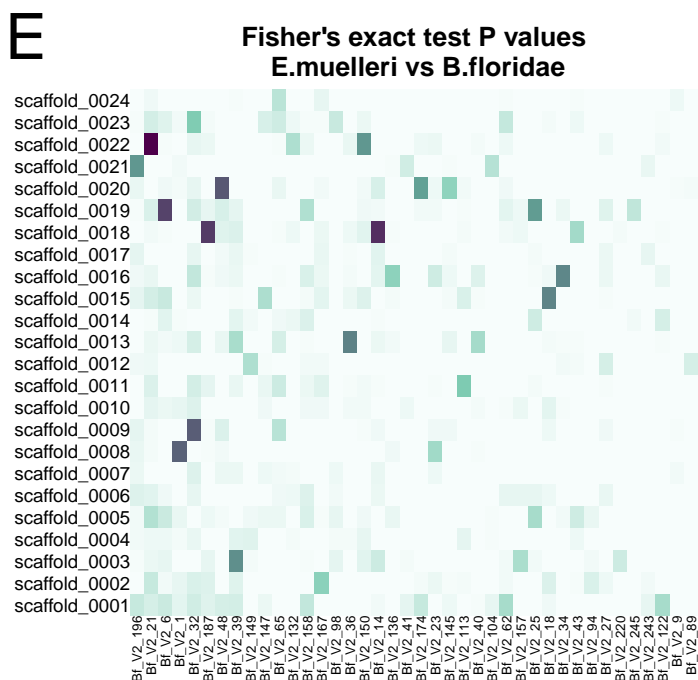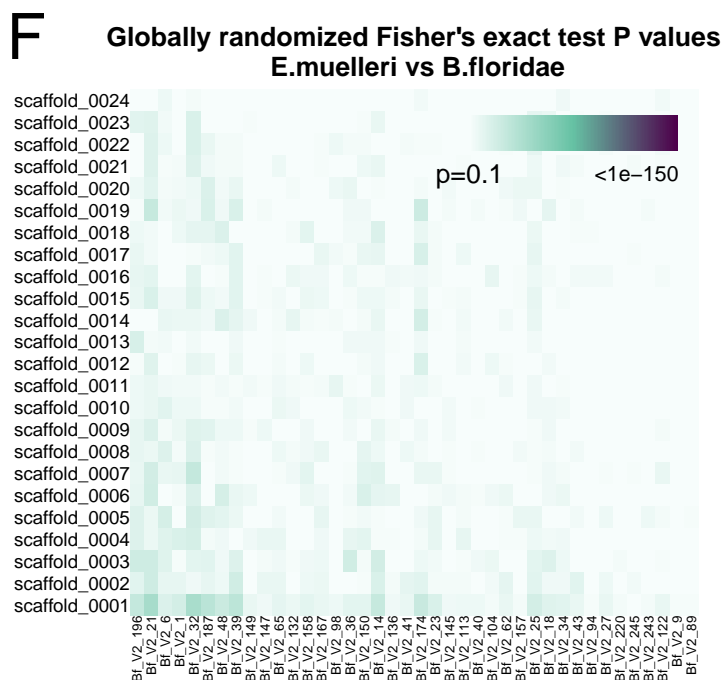

Supplement: Supplementary file 8 — Supplementary Data 4 [file 41467_2020_17397_MOESM8_ESM.zip › Supplementary_Data_4_Synteny_analyses_plots_scripts/fishers_test_graphs/ephydatia_vs_animals_ftest_pvalue_squares.pdf]

augustus\_sysnames\_prots\_vs\_Monosiga\_scaffold2D.tab

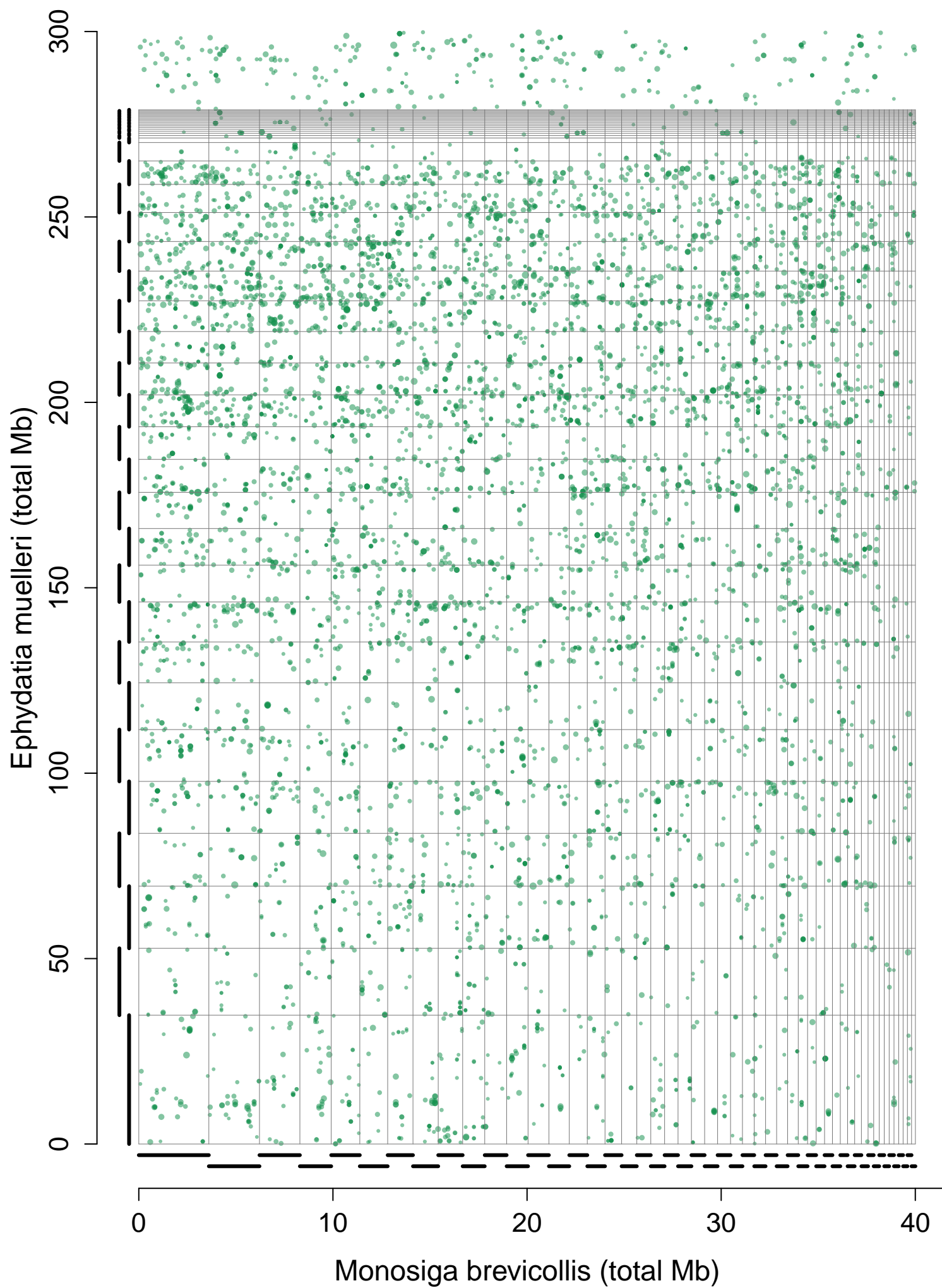

Supplement: Supplementary file 8 — Supplementary Data 4 [file 41467_2020_17397_MOESM8_ESM.zip › Supplementary_Data_4_Synteny_analyses_plots_scripts/emu_vs_choanos_supp_figs/prots_vs_Monosiga_scaffold2D.pdf]

augustus\_sysnames\_prots\_vs\_Srosetta\_scaffold2D.tab

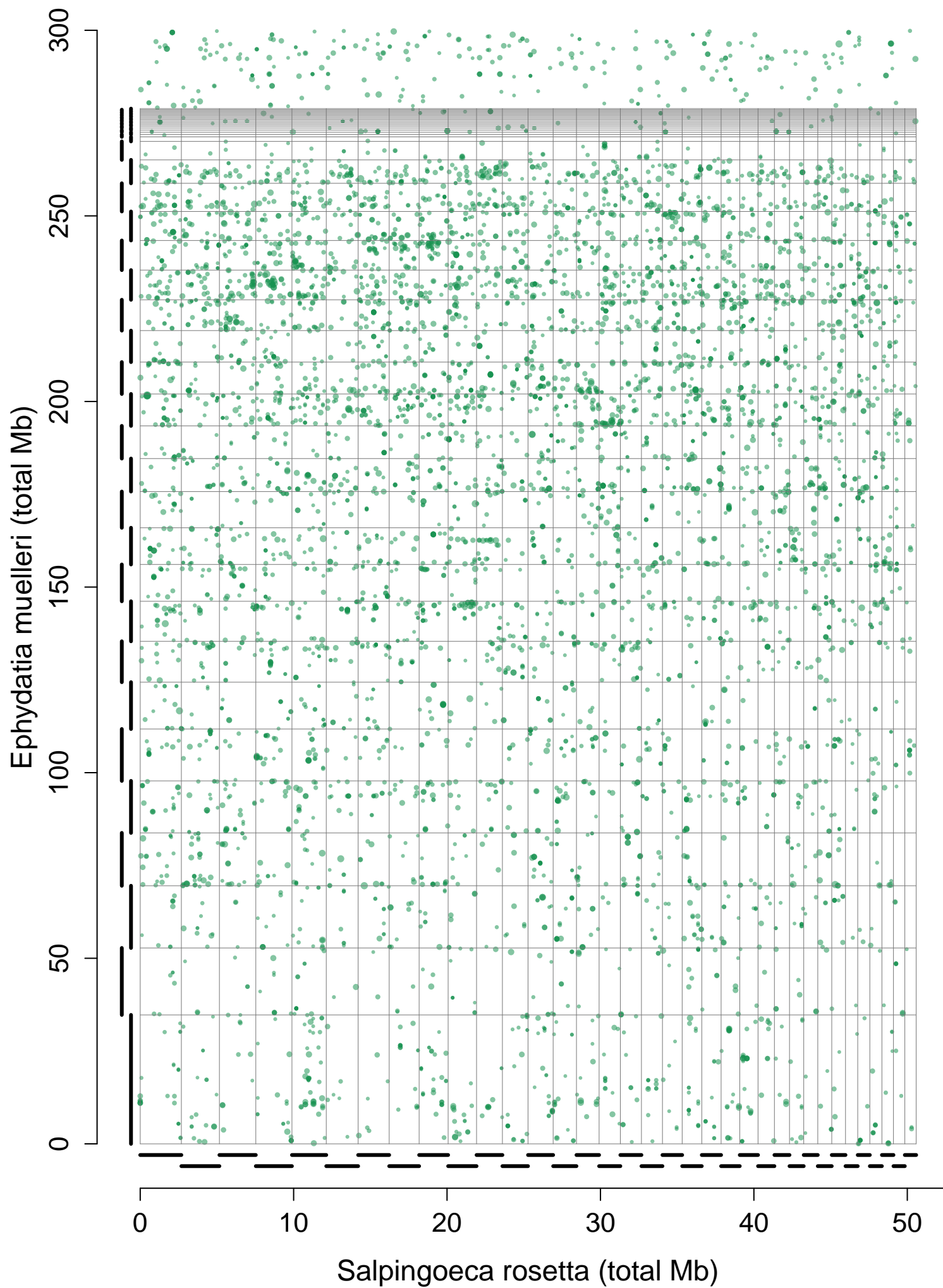

Supplement: Supplementary file 8 — Supplementary Data 4 [file 41467_2020_17397_MOESM8_ESM.zip › Supplementary_Data_4_Synteny_analyses_plots_scripts/emu_vs_choanos_supp_figs/prots_vs_Srosetta_scaffold2D.pdf]

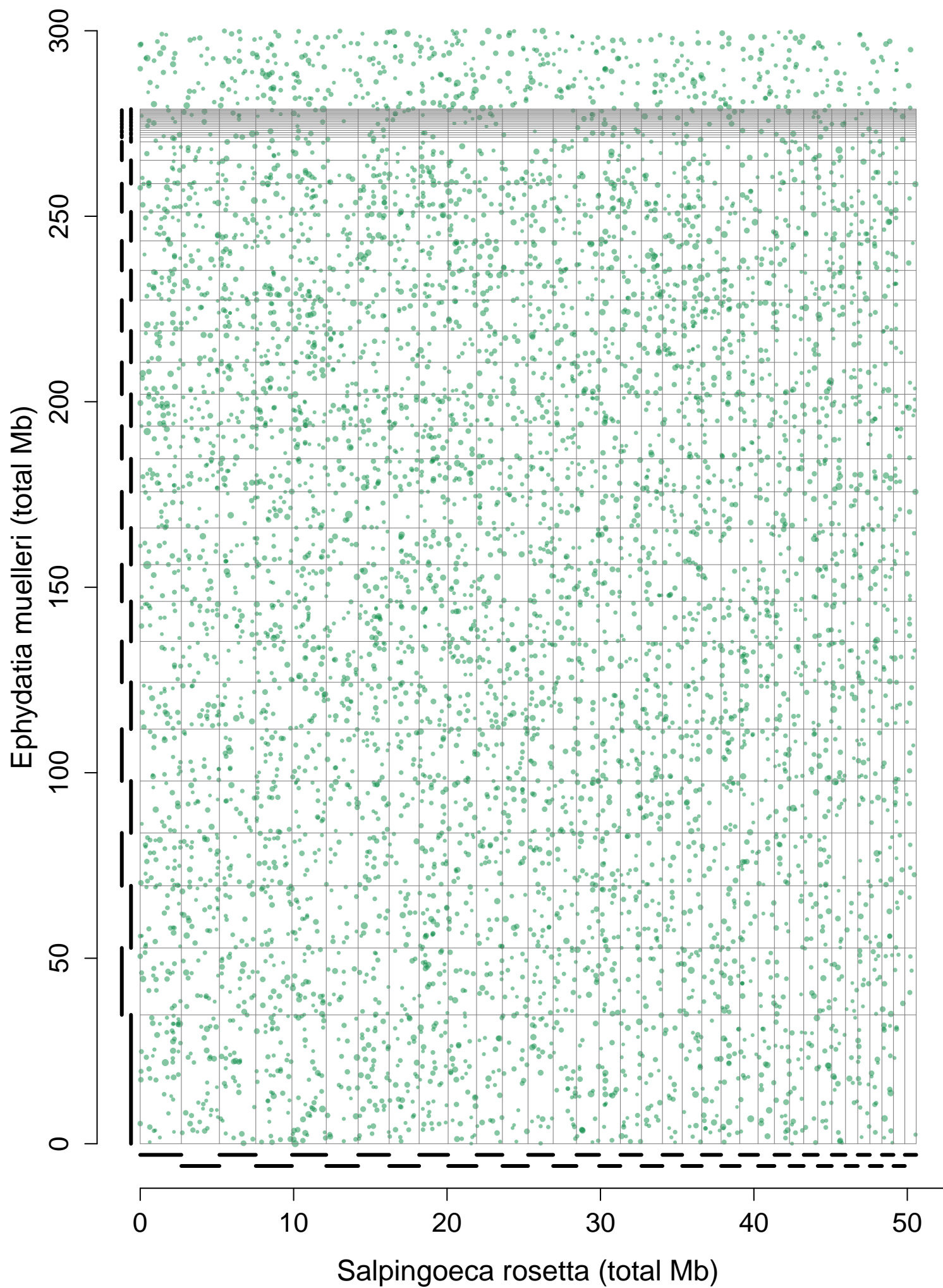

Supplement: Supplementary file 8 — Supplementary Data 4 [file 41467_2020_17397_MOESM8_ESM.zip › Supplementary_Data_4_Synteny_analyses_plots_scripts/emu_vs_choanos_supp_figs/prots_vs_Srosetta_scaffold2D_random.pdf]

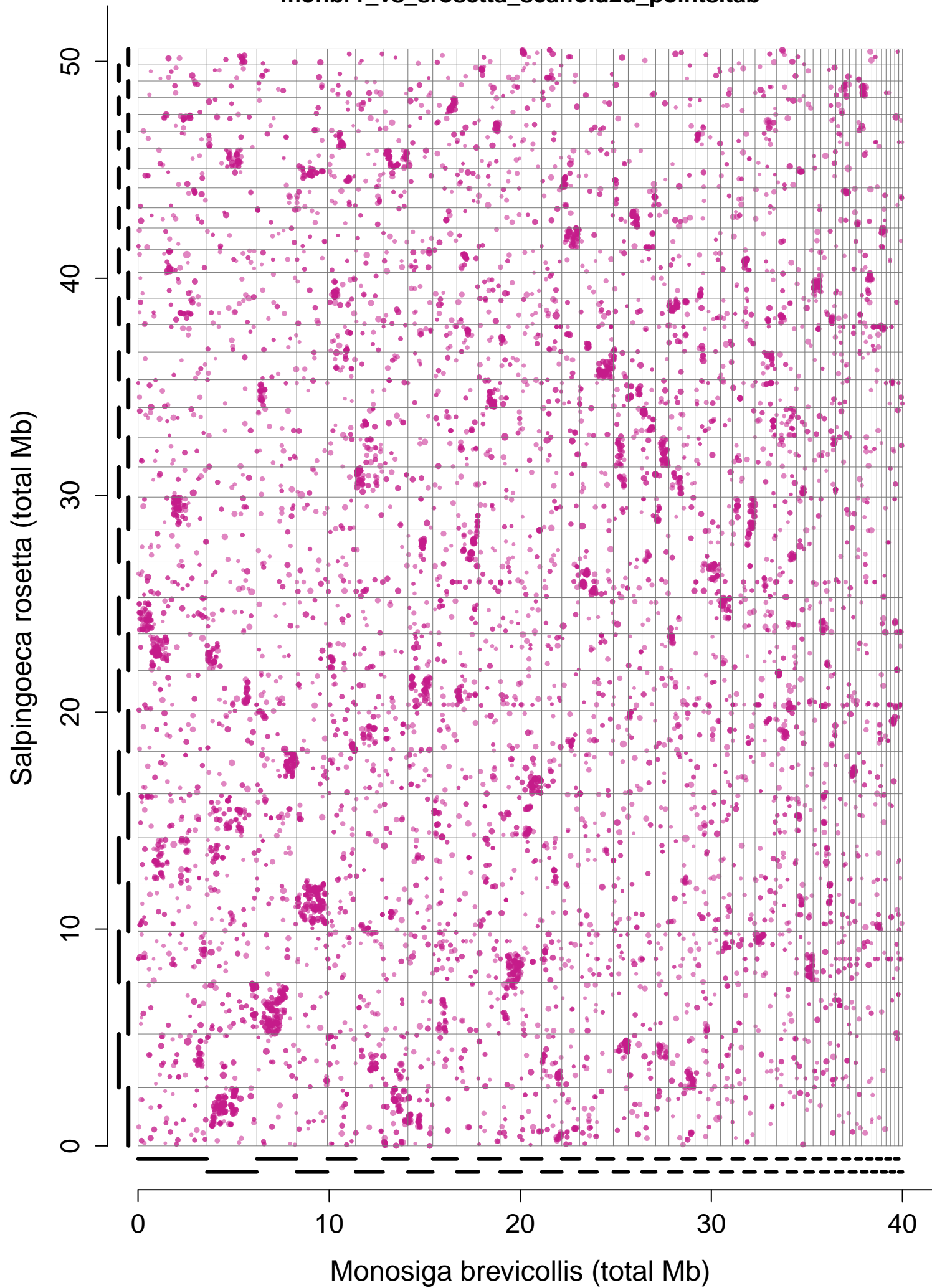

Supplement: Supplementary file 8 — Supplementary Data 4 [file 41467_2020_17397_MOESM8_ESM.zip › Supplementary_Data_4_Synteny_analyses_plots_scripts/emu_vs_choanos_supp_figs/monbr1_vs_srosetta_scaffold2d_points.pdf]

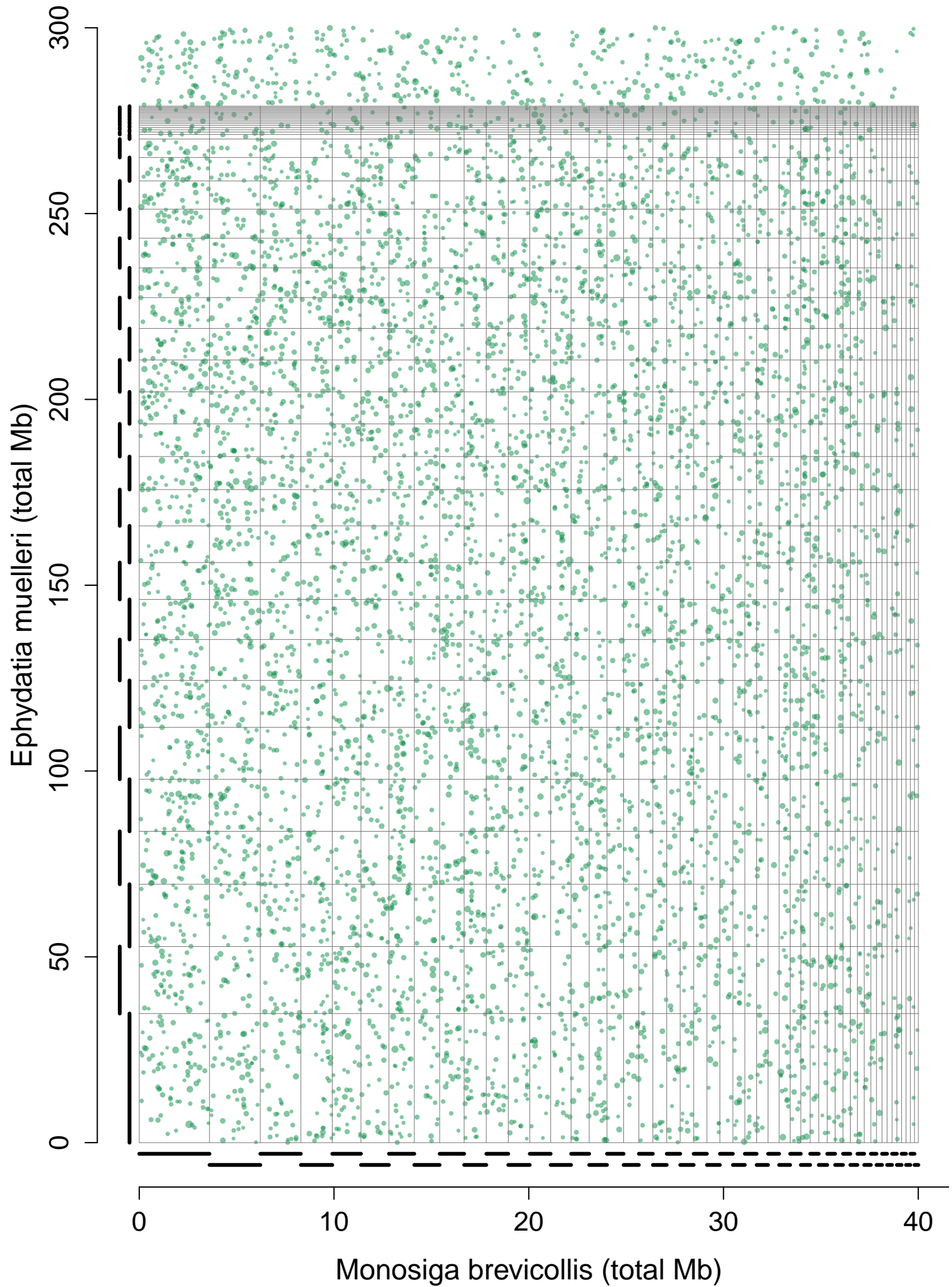

Supplement: Supplementary file 8 — Supplementary Data 4 [file 41467_2020_17397_MOESM8_ESM.zip › Supplementary_Data_4_Synteny_analyses_plots_scripts/emu_vs_choanos_supp_figs/prots_vs_Monosiga_scaffold2D_random.pdf]

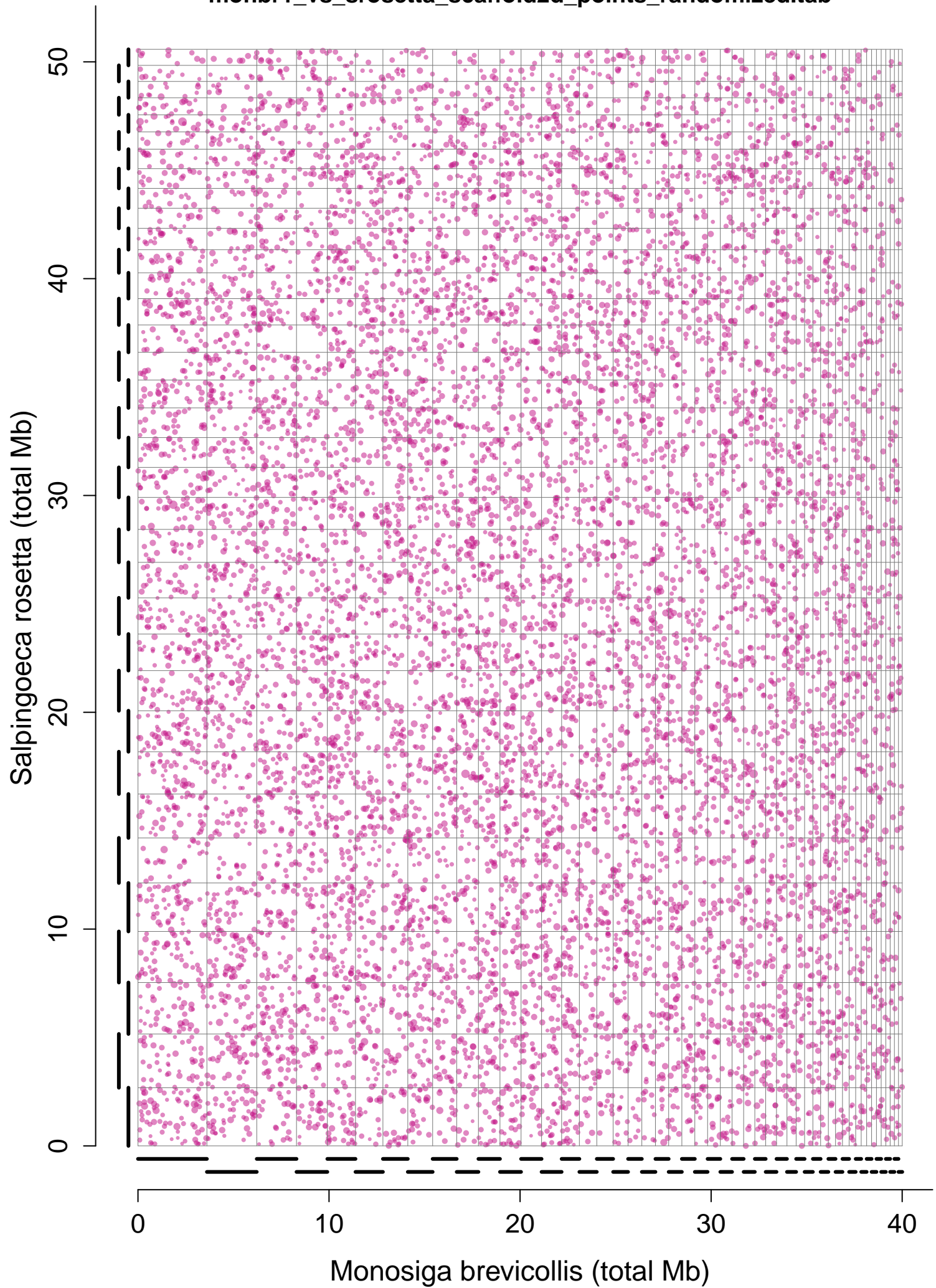

Supplement: Supplementary file 8 — Supplementary Data 4 [file 41467_2020_17397_MOESM8_ESM.zip › Supplementary_Data_4_Synteny_analyses_plots_scripts/emu_vs_choanos_supp_figs/monbr1_vs_srosetta_scaffold2d_points_rand.pdf]

augustus\_sysnames\_protvs\_vs\_Brafl2-AUG\_scaffold2D.tab

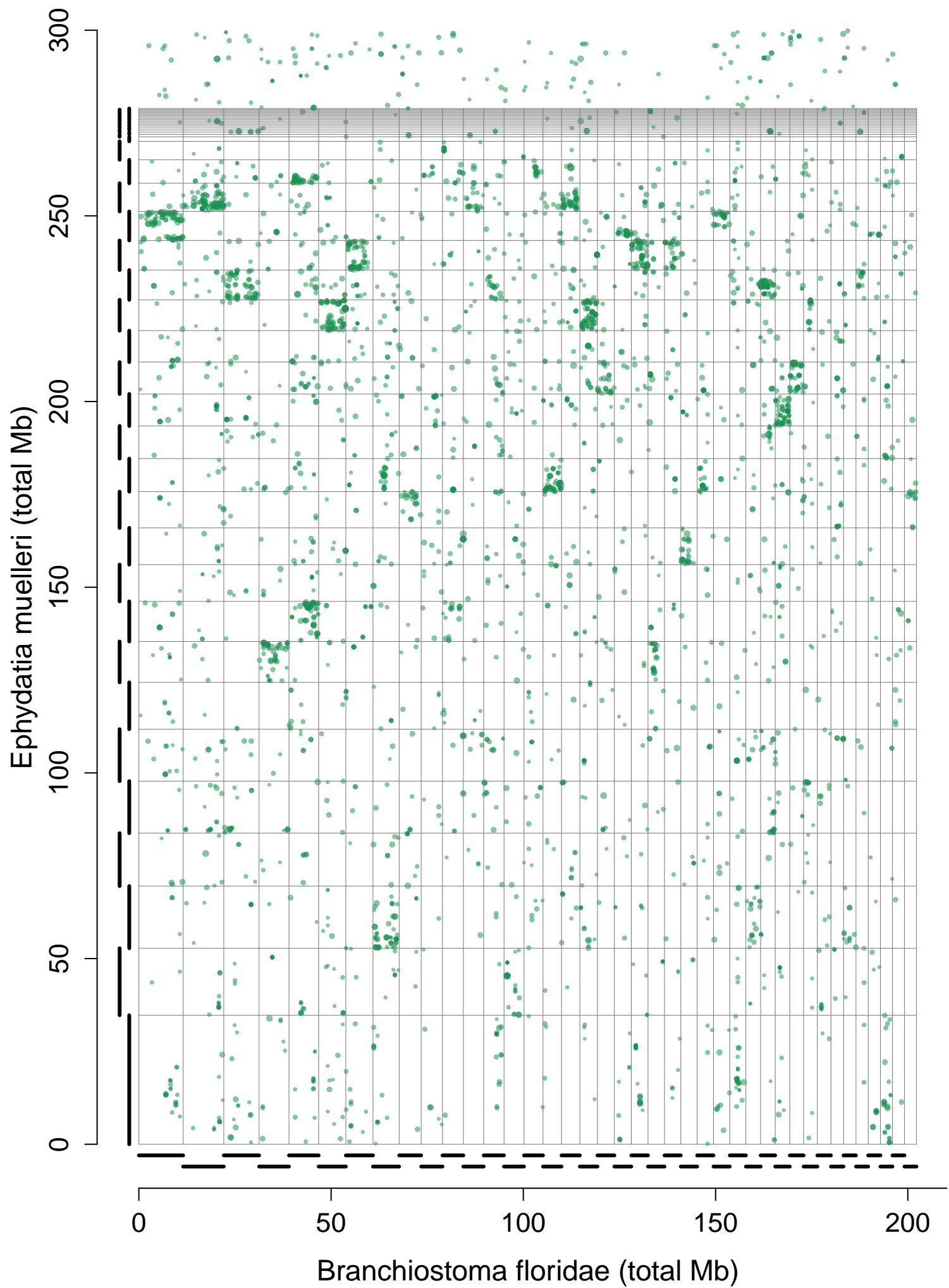

Supplement: Supplementary file 8 — Supplementary Data 4 [file 41467_2020_17397_MOESM8_ESM.zip › Supplementary_Data_4_Synteny_analyses_plots_scripts/emu_vs_animals_supp_figs/prots_vs_Brafl2-AUG_scaffold2D.pdf]

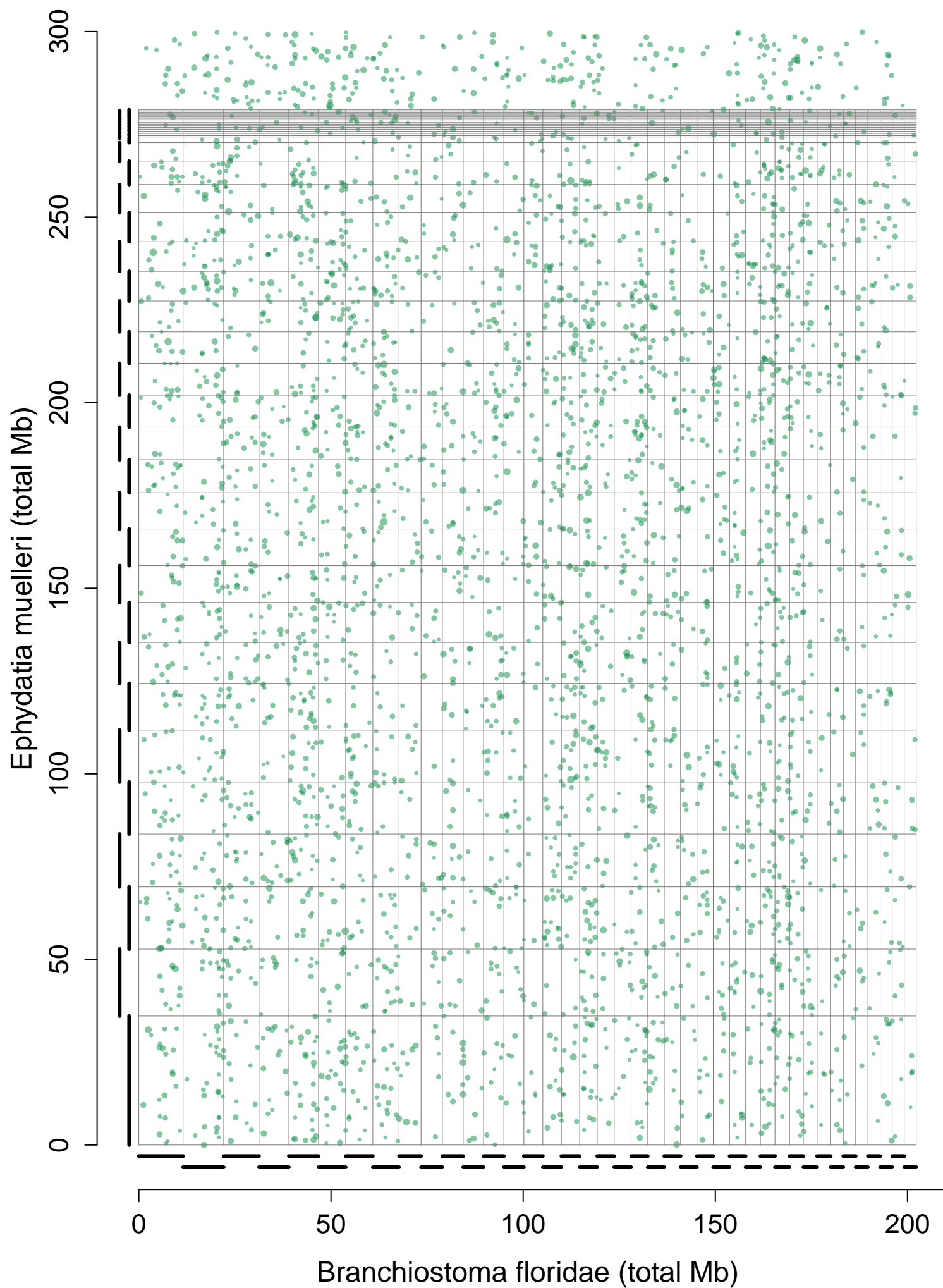

Supplement: Supplementary file 8 — Supplementary Data 4 [file 41467_2020_17397_MOESM8_ESM.zip › Supplementary_Data_4_Synteny_analyses_plots_scripts/emu_vs_animals_supp_figs/prots_vs_Brafl2-AUG_random_scaffold2D_test.pdf]

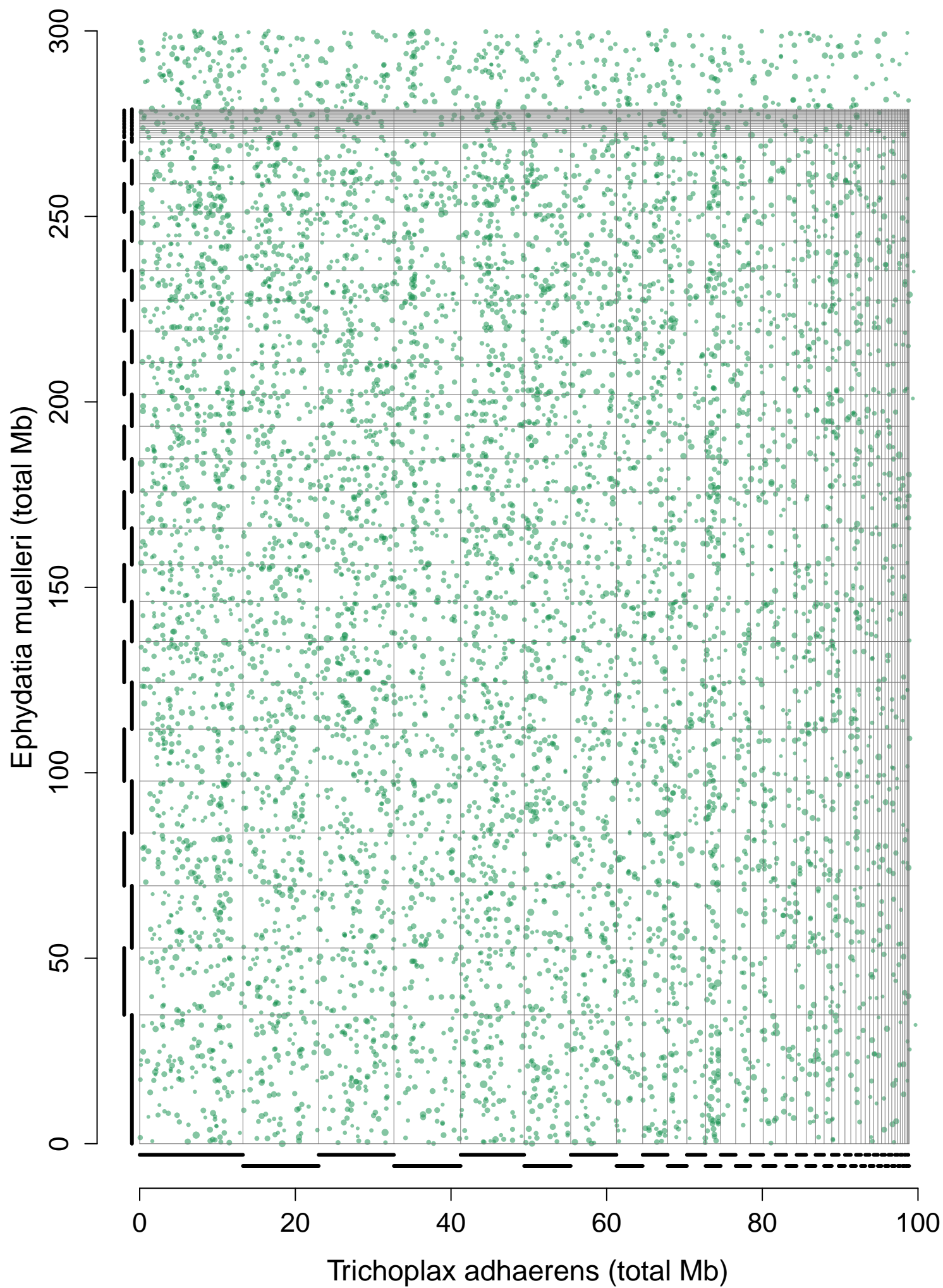

Supplement: Supplementary file 8 — Supplementary Data 4 [file 41467_2020_17397_MOESM8_ESM.zip › Supplementary_Data_4_Synteny_analyses_plots_scripts/emu_vs_animals_supp_figs/prots_vs_Triad1_scaffold2D_random.pdf]

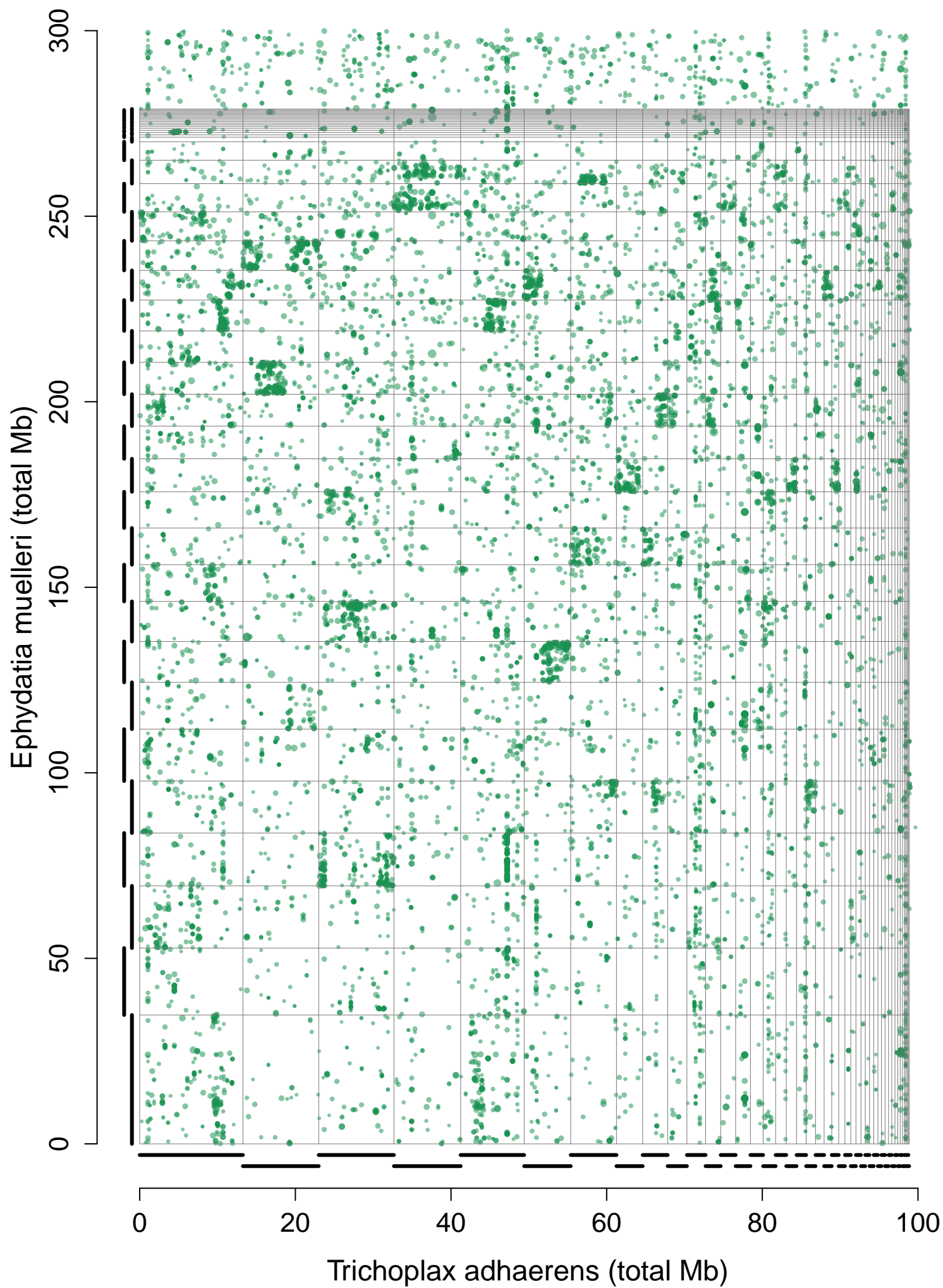

Supplement: Supplementary file 8 — Supplementary Data 4 [file 41467_2020_17397_MOESM8_ESM.zip › Supplementary_Data_4_Synteny_analyses_plots_scripts/emu_vs_animals_supp_figs/prots_vs_Triad1_scaffold2D.pdf]

augustus\_sysnames\_prots\_vs\_Nve2\_scaffold2D.tab

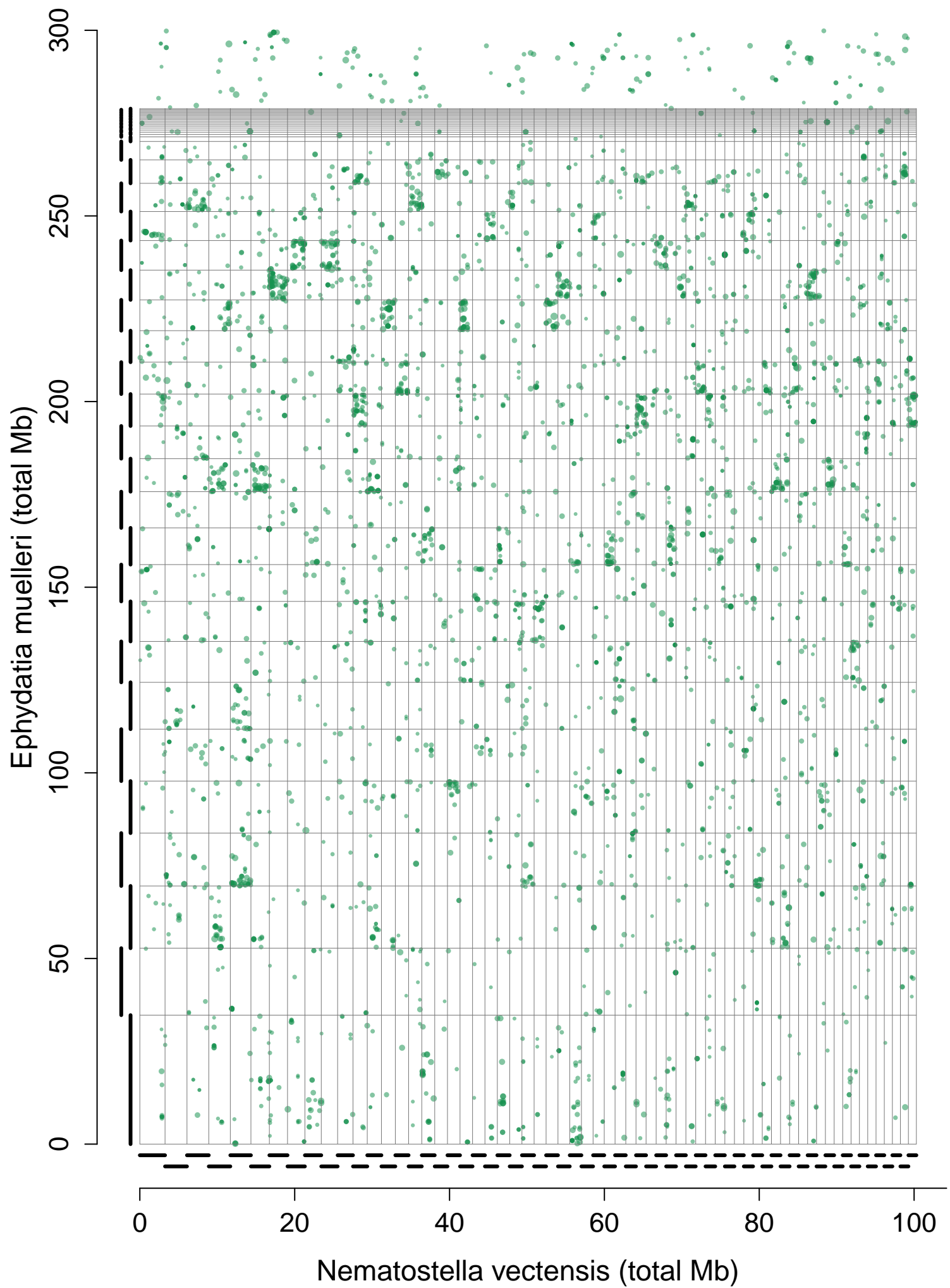

Supplement: Supplementary file 8 — Supplementary Data 4 [file 41467_2020_17397_MOESM8_ESM.zip › Supplementary_Data_4_Synteny_analyses_plots_scripts/emu_vs_animals_supp_figs/prots_vs_Nve2_scaffold2D.pdf]

augustus\_sysnames\_prots\_vs\_Nve2\_scaffold2D\_random.tab

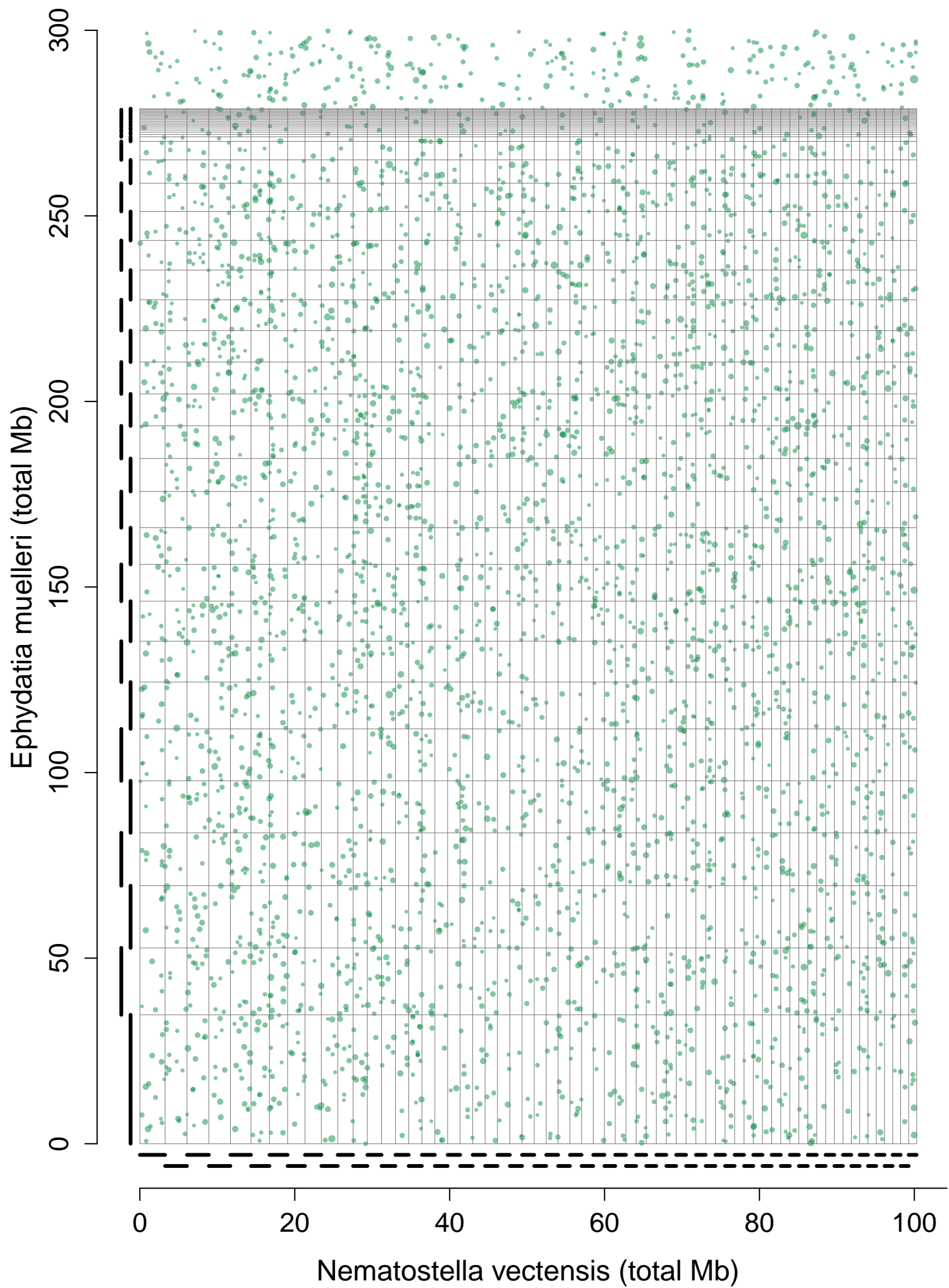

Supplement: Supplementary file 8 — Supplementary Data 4 [file 41467_2020_17397_MOESM8_ESM.zip › Supplementary_Data_4_Synteny_analyses_plots_scripts/emu_vs_animals_supp_figs/prots_vs_Nve2_scaffold2D_random.pdf]

*Ephydatia muelleri*

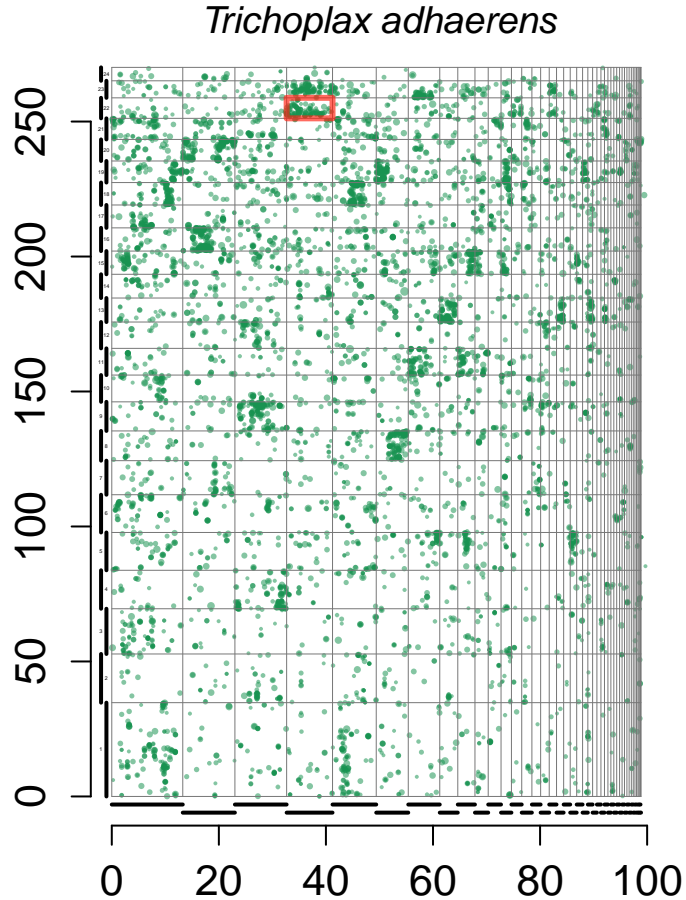

Length (Mb)

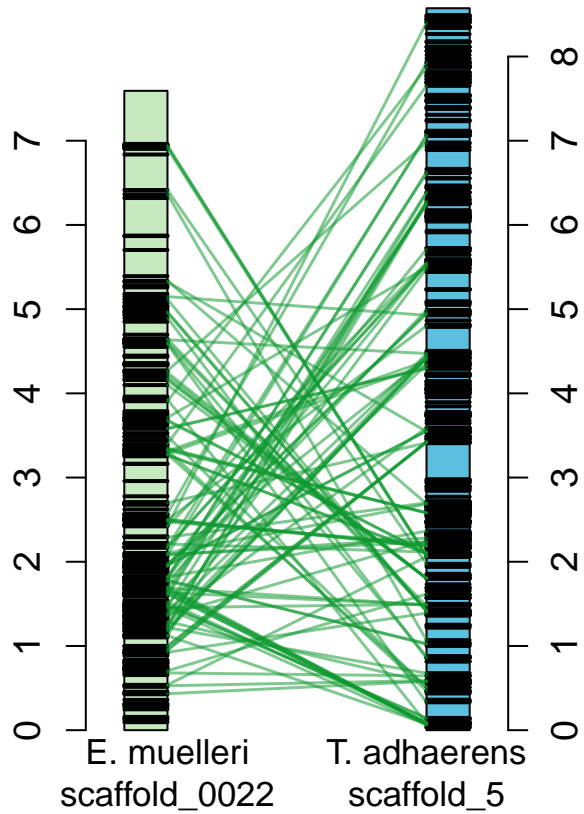

Supplement: Supplementary file 8 — Supplementary Data 4 [file 41467_2020_17397_MOESM8_ESM.zip › Supplementary_Data_4_Synteny_analyses_plots_scripts/figures/ephydatia_Ta_synteny_dot_plots_w_chr22.pdf]

*Branchiostoma floridae*

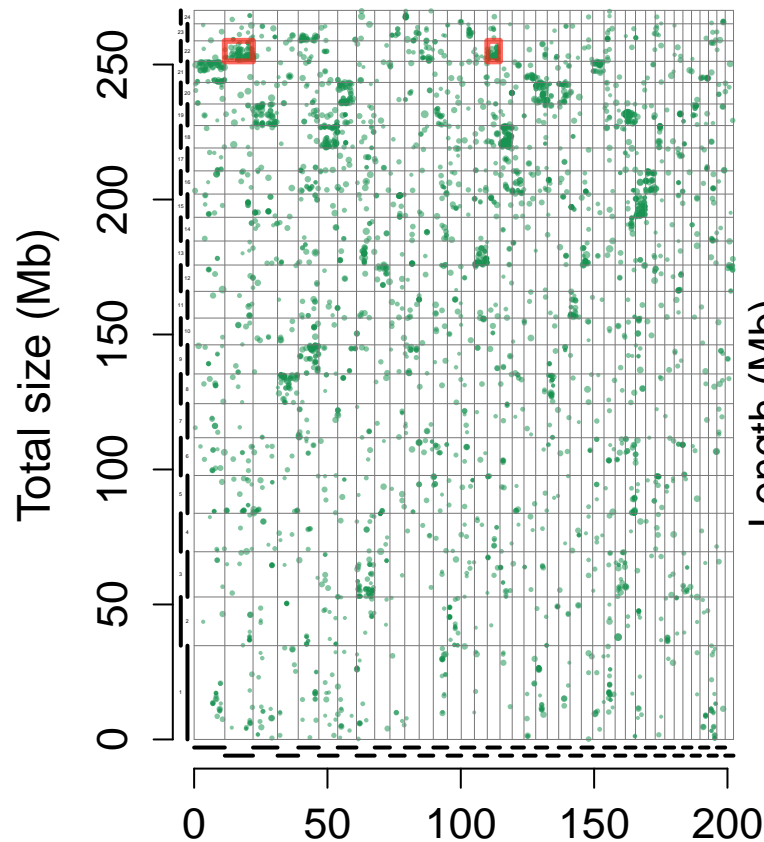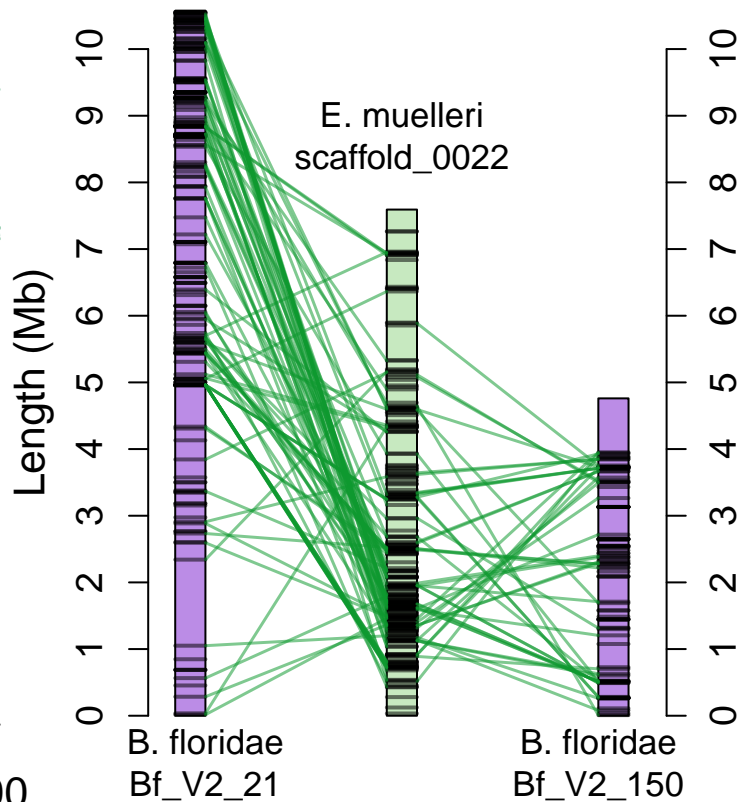

Supplement: Supplementary file 8 — Supplementary Data 4 [file 41467_2020_17397_MOESM8_ESM.zip › Supplementary_Data_4_Synteny_analyses_plots_scripts/figures/ephydatia_Bf_synteny_dot_plots_w_chr22.pdf]

hoilungia\_vs\_trichoplax\_scaffold2d\_points\_g-random.tab

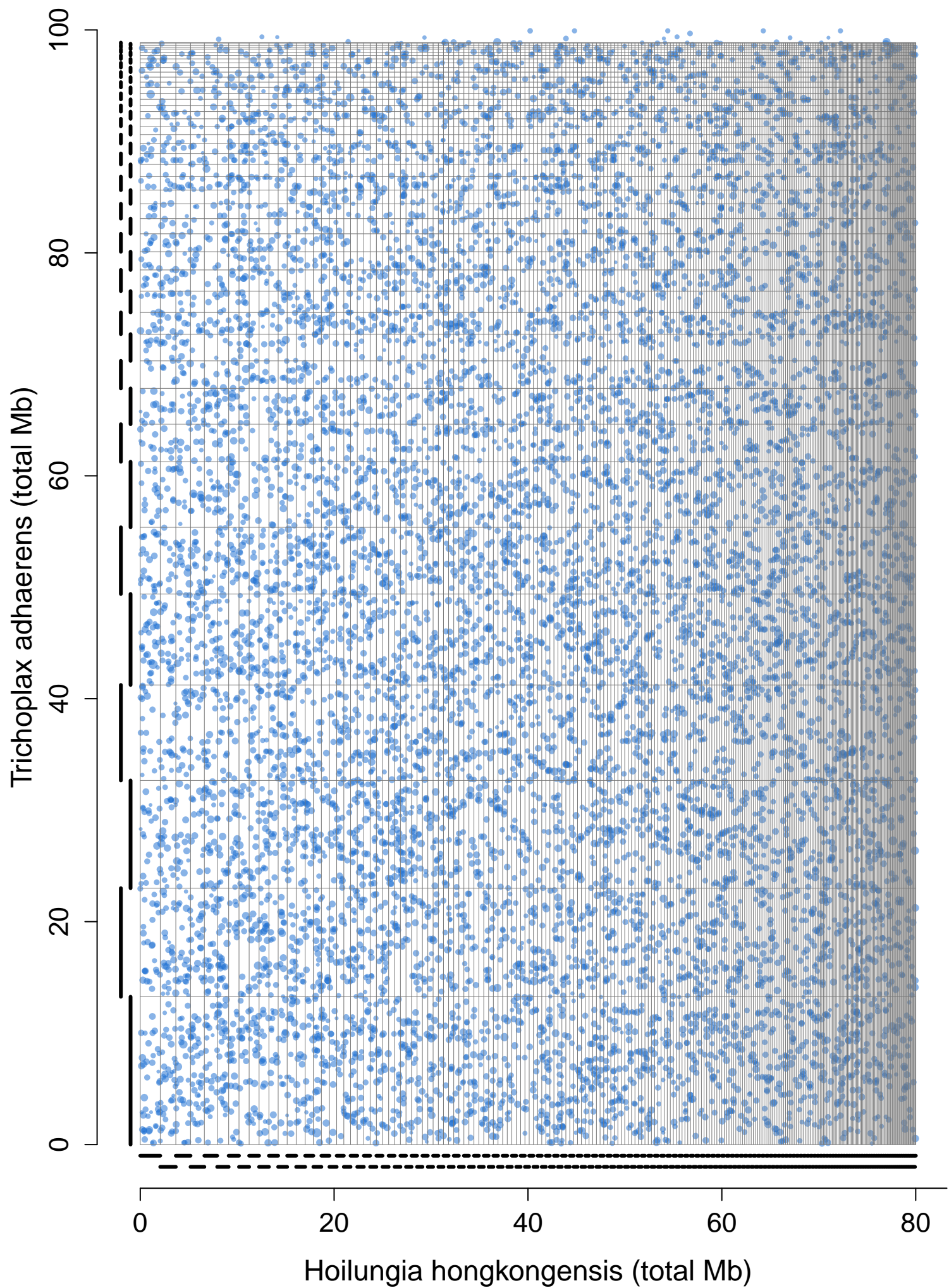

Supplement: Supplementary file 8 — Supplementary Data 4 [file 41467_2020_17397_MOESM8_ESM.zip › Supplementary_Data_4_Synteny_analyses_plots_scripts/placozoan_comparison/hoilungia_vs_trichoplax_scaf2d_points_g-random.pdf]

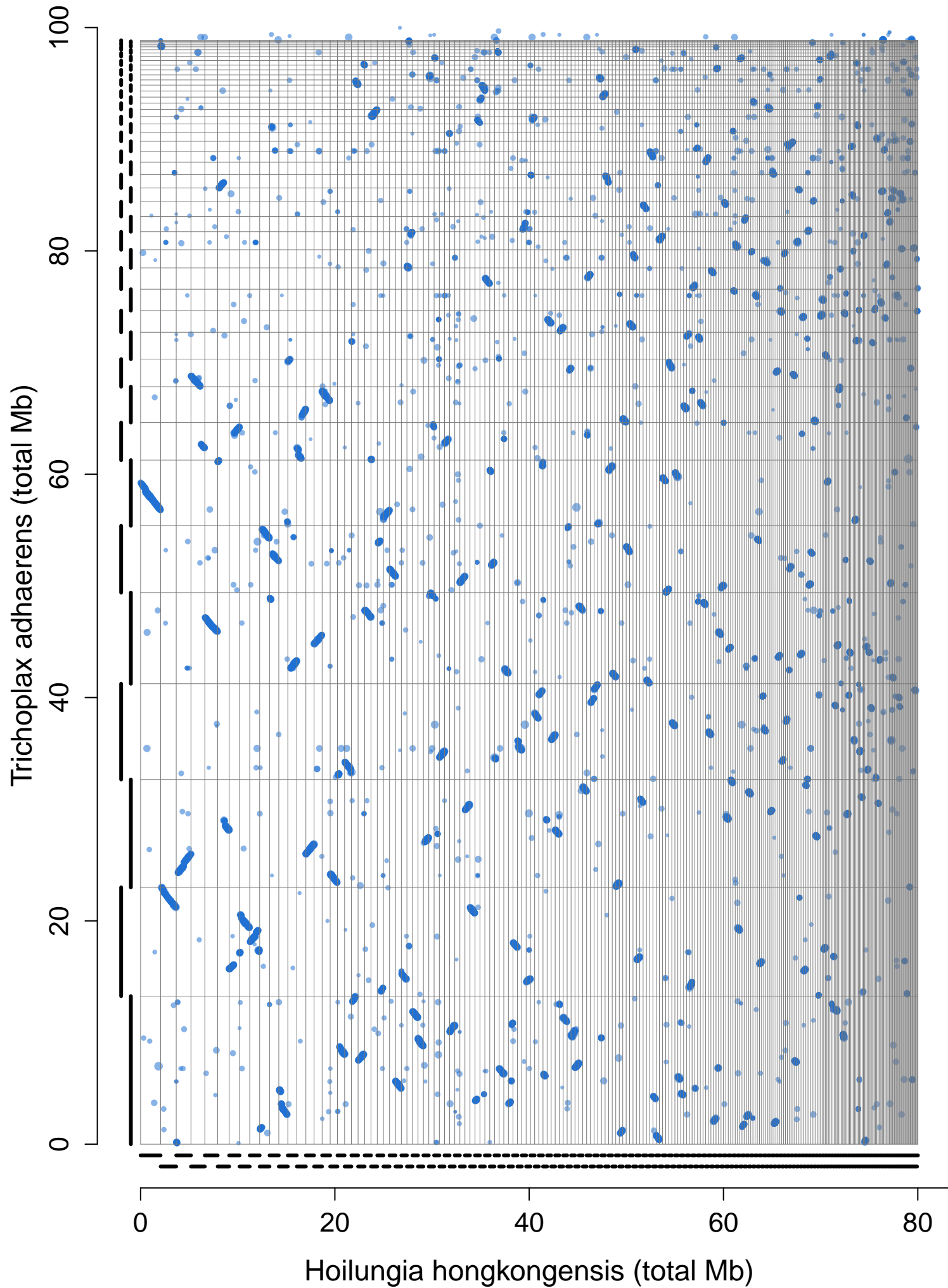

Supplement: Supplementary file 8 — Supplementary Data 4 [file 41467_2020_17397_MOESM8_ESM.zip › Supplementary_Data_4_Synteny_analyses_plots_scripts/placozoan_comparison/hoilungia_vs_trichoplax_scaf2d_points.pdf]

hoilungia\_vs\_trichoplax\_scaffold2d\_points\_s-random.tab

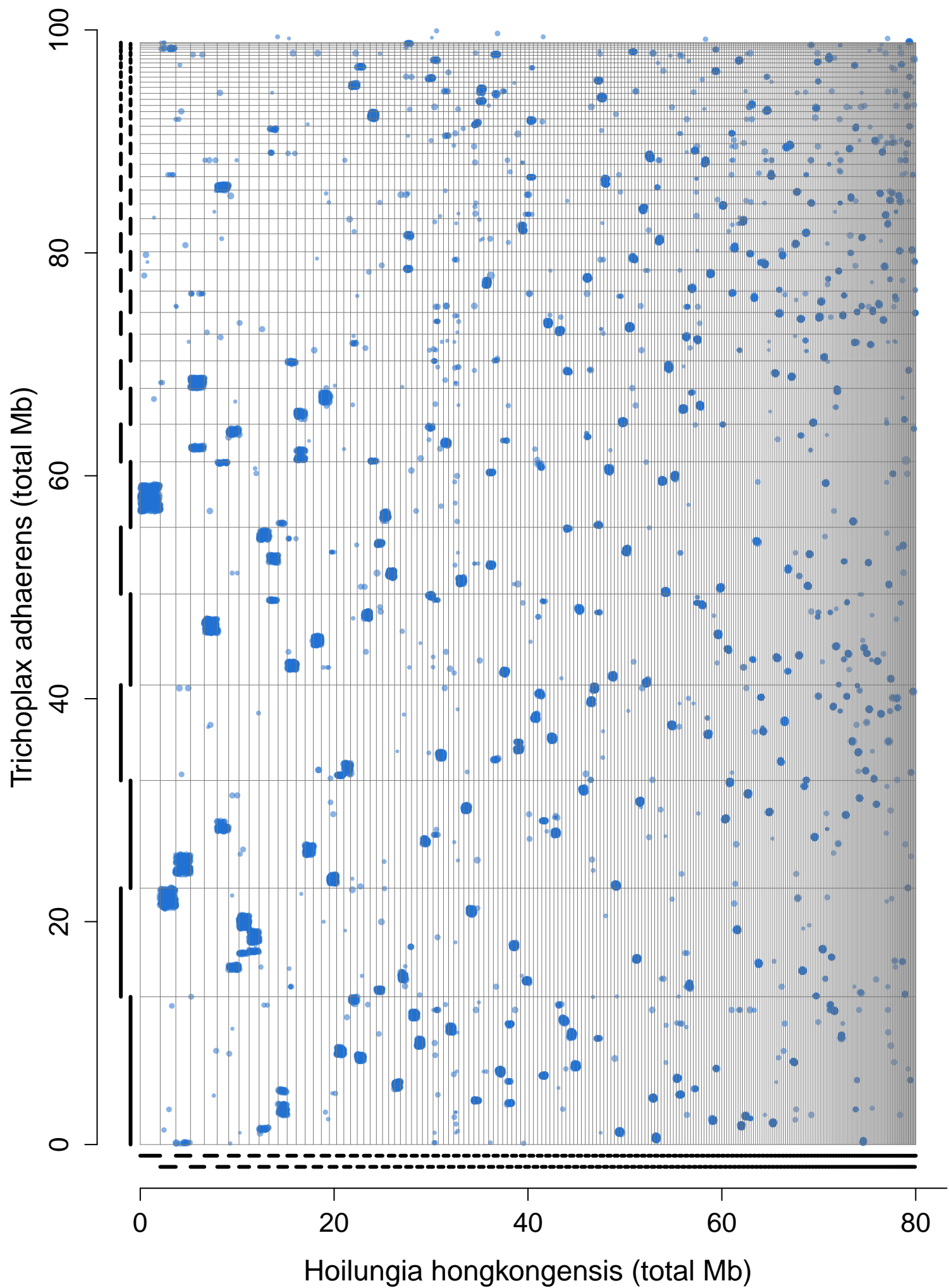

Supplement: Supplementary file 8 — Supplementary Data 4 [file 41467_2020_17397_MOESM8_ESM.zip › Supplementary_Data_4_Synteny_analyses_plots_scripts/placozoan_comparison/hoilungia_vs_trichoplax_scaf2d_points_s-random.pdf]

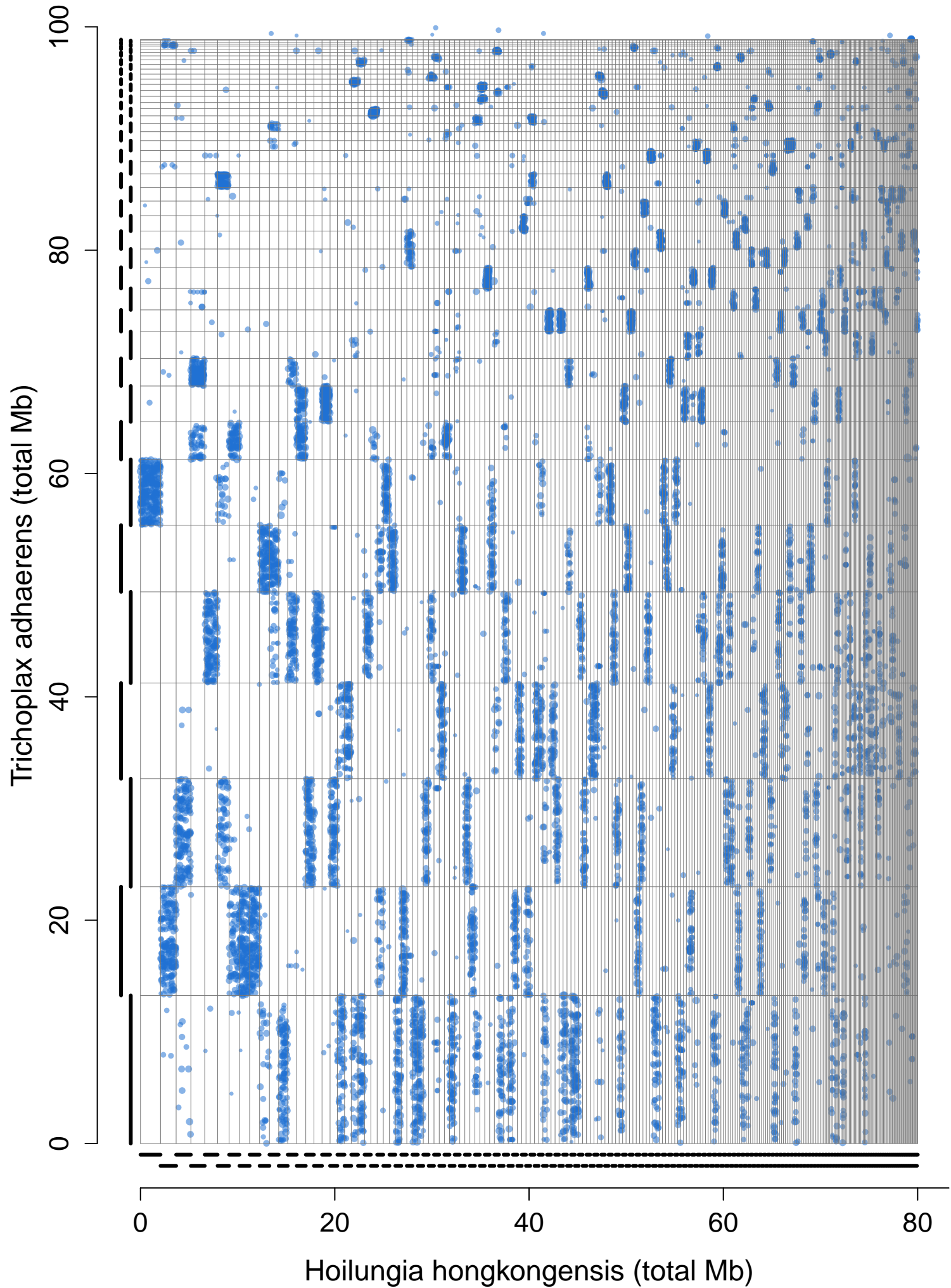

Supplement: Supplementary file 8 — Supplementary Data 4 [file 41467_2020_17397_MOESM8_ESM.zip › Supplementary_Data_4_Synteny_analyses_plots_scripts/placozoan_comparison/hoilungia_vs_trichoplax_scaf2d_points_ds-random.pdf]

REVIGO Gene Ontology treemap

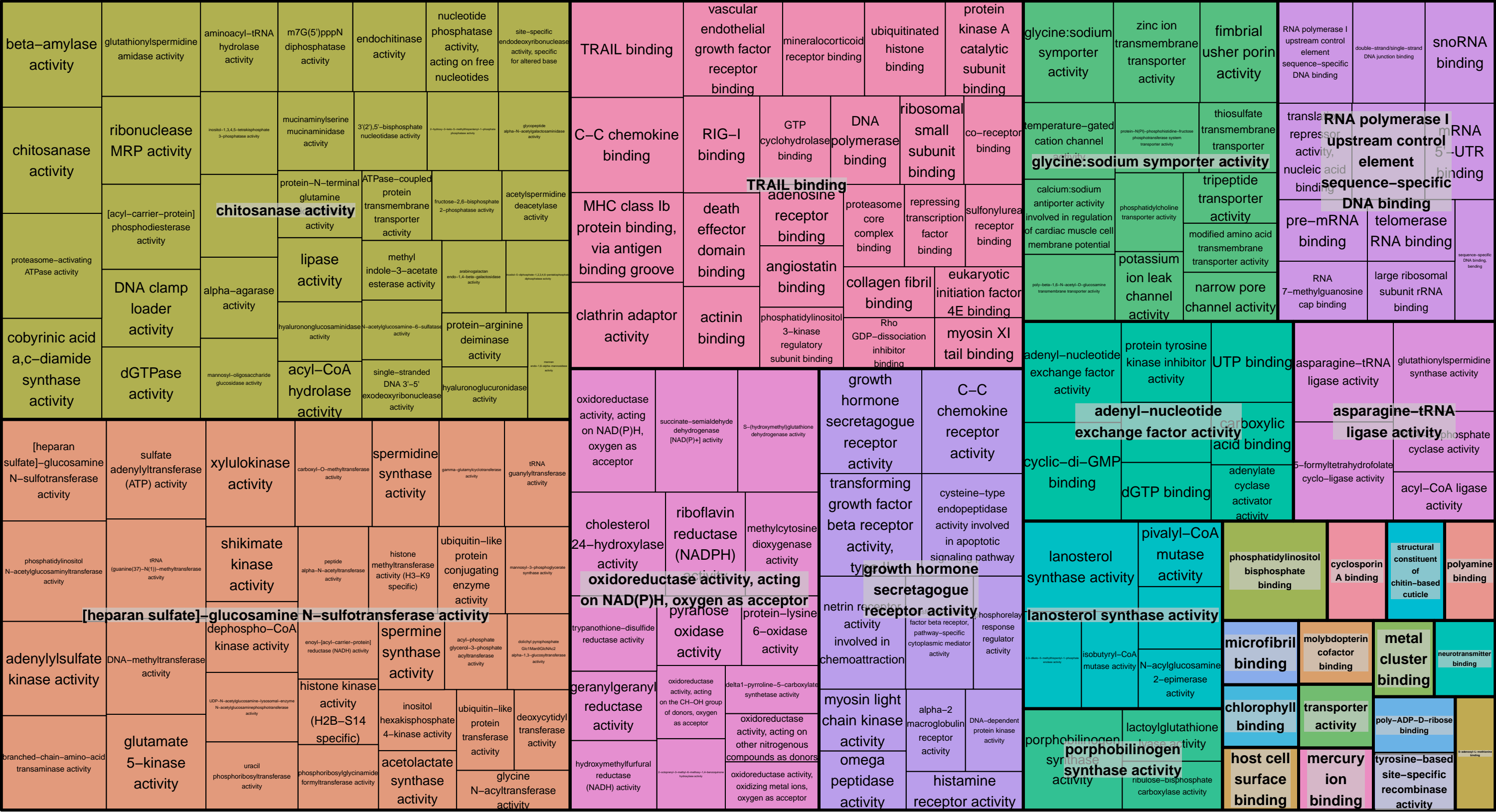

Supplement: Supplementary file 11 — Supplementary Data 7 [file 41467_2020_17397_MOESM11_ESM.zip › Supplementary_Data_7_Clustering_analyses_full_lists/Supp_Data_7H_Figures_EPMU_REVIGO_treemap_over_all_GO_terms_MF_3.pdf]

REVIGO Gene Ontology treemap

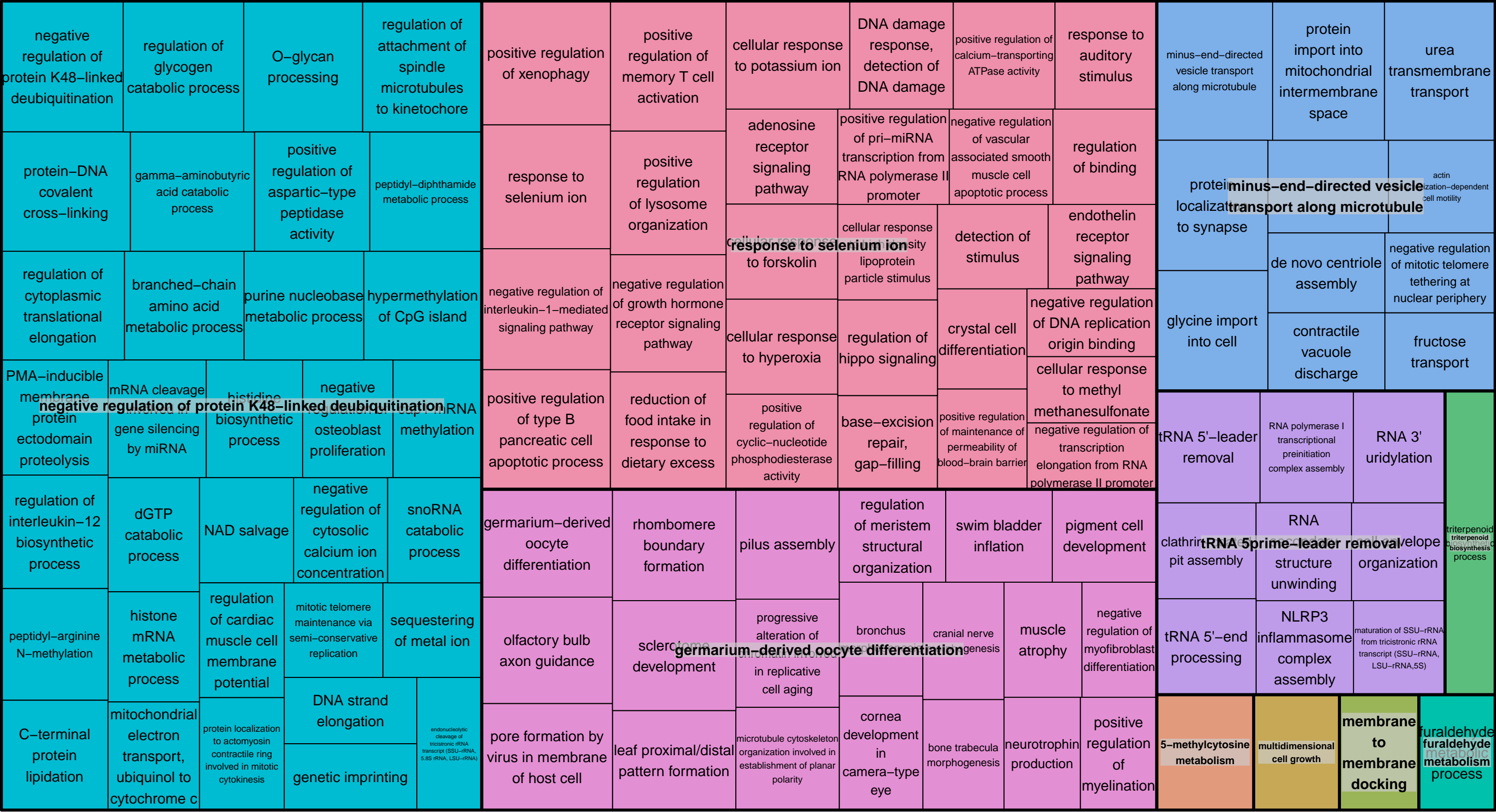

Supplement: Supplementary file 11 — Supplementary Data 7 [file 41467_2020_17397_MOESM11_ESM.zip › Supplementary_Data_7_Clustering_analyses_full_lists/Supp_Data_7E_Figures_EPMU_REVIGO_treemap_for_over_all_GO_terms_BP.pdf]

## **E. muelleri**

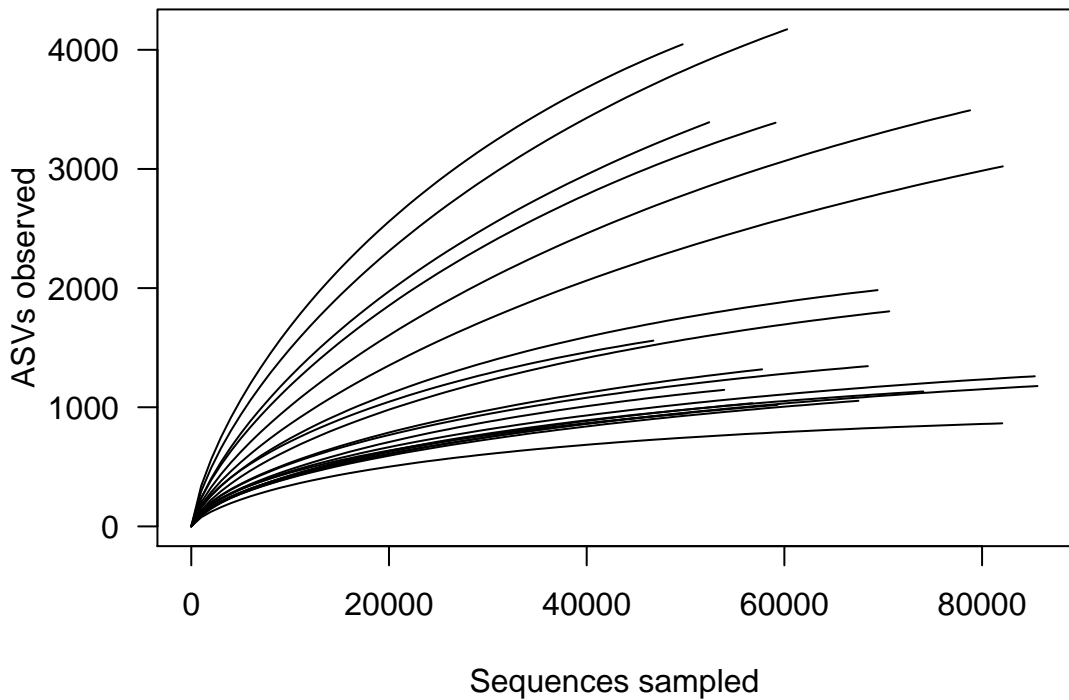

Supplement: Supplementary file 13 — Supplementary Data 9 [file 41467_2020_17397_MOESM13_ESM.zip › Supplementary_Data_9_Amplicon_seq_raw_results/Supplementary_Data_9A_rarefaction.pdf]
