# Supplementary material for: Tracing animal genomic evolution with the chromosomal-level assembly of the freshwater sponge Ephydatia muelleri
Source: Nat Commun. 2020 Jul 27;11:3676. doi: 10.1038/s41467-020-17397-w (PMC7385117; doi:10.1038/s41467-020-17397-w)
Supplement: Supplementary file 8 — Supplementary Data 4 [file 41467_2020_17397_MOESM8_ESM.zip › Supplementary_Data_4_Synteny_analyses_plots_scripts/fishers_test_graphs/hoilungia_vs_trichoplax_ftest_pvalue_squares.pdf]

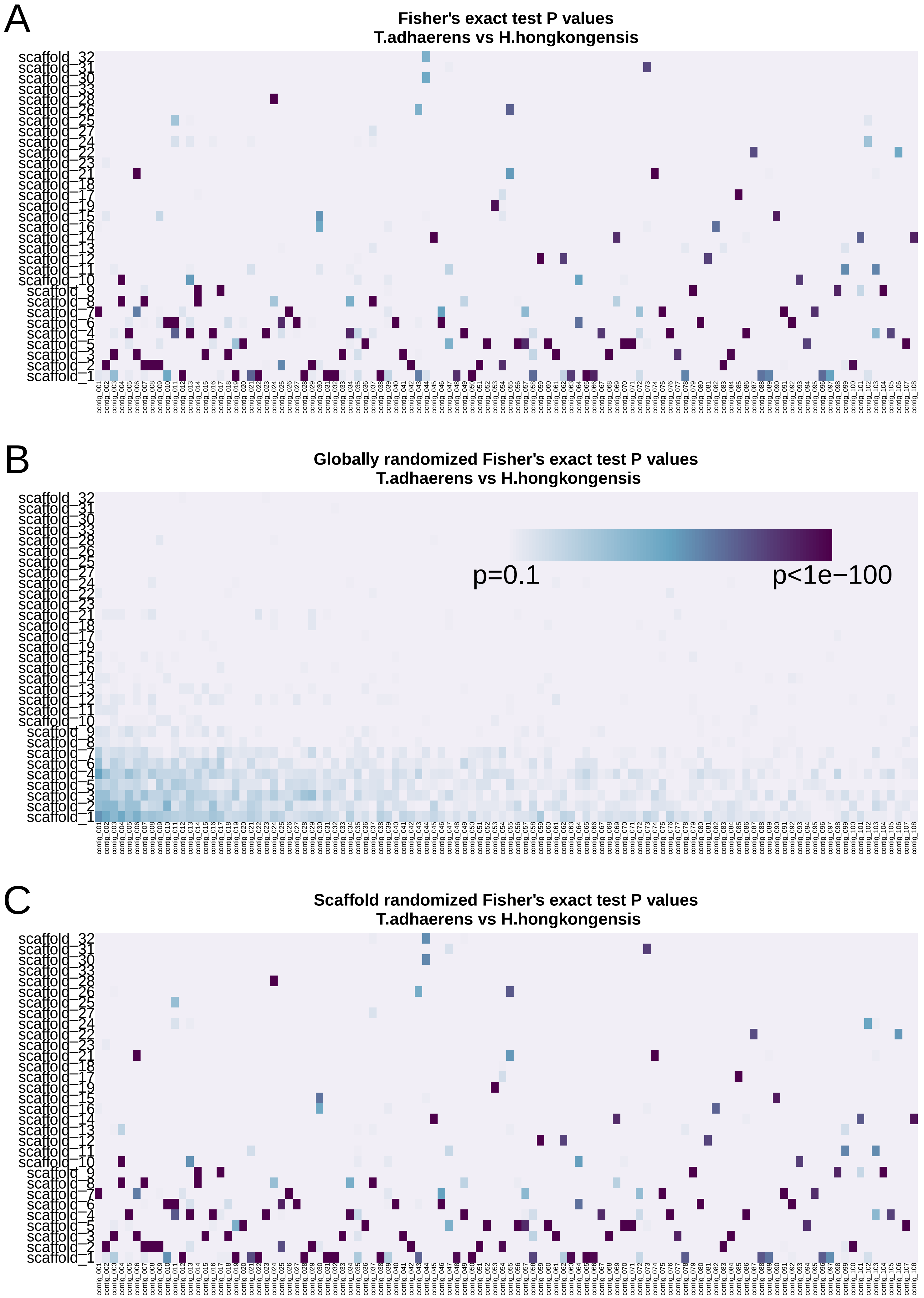
A
Fisher's exact test P values
T.adhaerens vs H.hongkongensis
B
Globally randomized Fisher's exact test P values
T.adhaerens vs H.hongkongensis
p=0.1
p<1e−100
C
Scaffold randomized Fisher's exact test P values
T.adhaerens vs H.hongkongensis
