# Supplementary material for: Tracing animal genomic evolution with the chromosomal-level assembly of the freshwater sponge Ephydatia muelleri
Source: Nat Commun. 2020 Jul 27;11:3676. doi: 10.1038/s41467-020-17397-w (PMC7385117; doi:10.1038/s41467-020-17397-w)
Supplement: Supplementary file 8 — Supplementary Data 4 [file 41467_2020_17397_MOESM8_ESM.zip › Supplementary_Data_4_Synteny_analyses_plots_scripts/fishers_test_graphs/choanoflagellates_ftest_pvalue_squares.pdf]

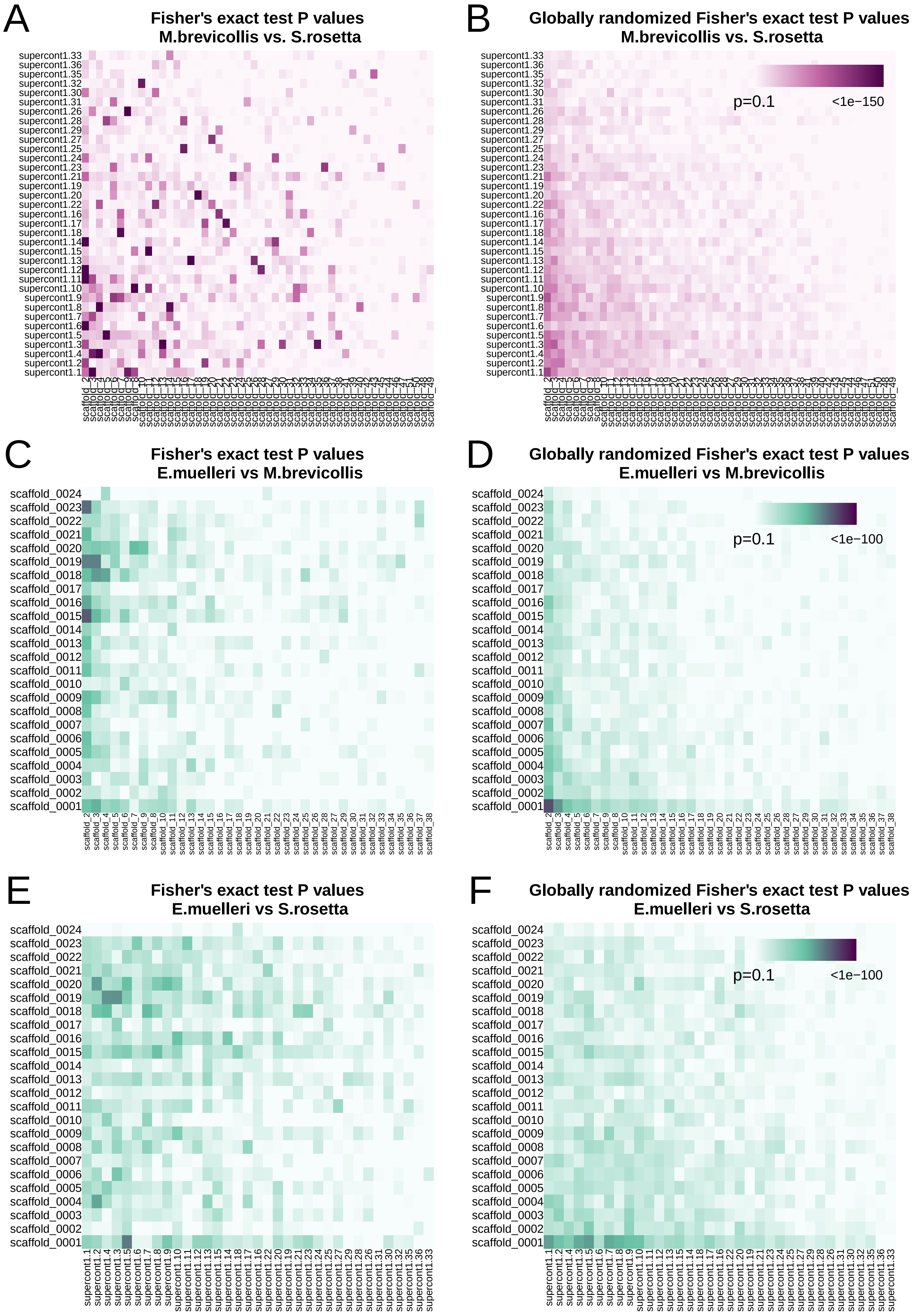
A
Fisher's exact test P values
M.brevicollis vs. S.rosetta
supercont1.33
supercont1.36
supercont1.35
supercont1.32
supercont1.30
supercont1.31
supercont1.26
supercont1.28
supercont1.29
supercont1.27
supercont1.25
supercont1.24
supercont1.23
supercont1.21
supercont1.19
supercont1.20
supercont1.22
supercont1.16
supercont1.17
supercont1.18
supercont1.14
supercont1.15
supercont1.13
supercont1.12
supercont1.11
supercont1.10
supercont1.9
supercont1.8
supercont1.7
supercont1.6
supercont1.5
supercont1.3
supercont1.4
supercont1.2
supercont1.1
B
Globally randomized Fisher's exact test P values
M.brevicollis vs. S.rosetta
p=0.1
<1e−150
C
Fisher's exact test P values
E.muelleri vs M.brevicollis
scaffold_0024
scaffold_0023
scaffold_0022
scaffold_0021
scaffold_0020
scaffold_0019
scaffold_0018
scaffold_0017
scaffold_0016
scaffold_0015
scaffold_0014
scaffold_0013
scaffold_0012
scaffold_0011
scaffold_0010
scaffold_0009
scaffold_0008
scaffold_0007
scaffold_0006
scaffold_0005
scaffold_0004
scaffold_0003
scaffold_0002
scaffold_0001
D
Globally randomized Fisher's exact test P values
E.muelleri vs M.brevicollis
p=0.1
<1e−100
E
Fisher's exact test P values
E.muelleri vs S.rosetta
F
Globally randomized Fisher's exact test P values
E.muelleri vs S.rosetta
p=0.1
<1e−100
