# Supplementary material for: Tracing animal genomic evolution with the chromosomal-level assembly of the freshwater sponge Ephydatia muelleri
Source: Nat Commun. 2020 Jul 27;11:3676. doi: 10.1038/s41467-020-17397-w (PMC7385117; doi:10.1038/s41467-020-17397-w)
Supplement: Supplementary file 8 — Supplementary Data 4 [file 41467_2020_17397_MOESM8_ESM.zip › Supplementary_Data_4_Synteny_analyses_plots_scripts/figures/ephydatia_combined_synteny_dot_plots.pdf]

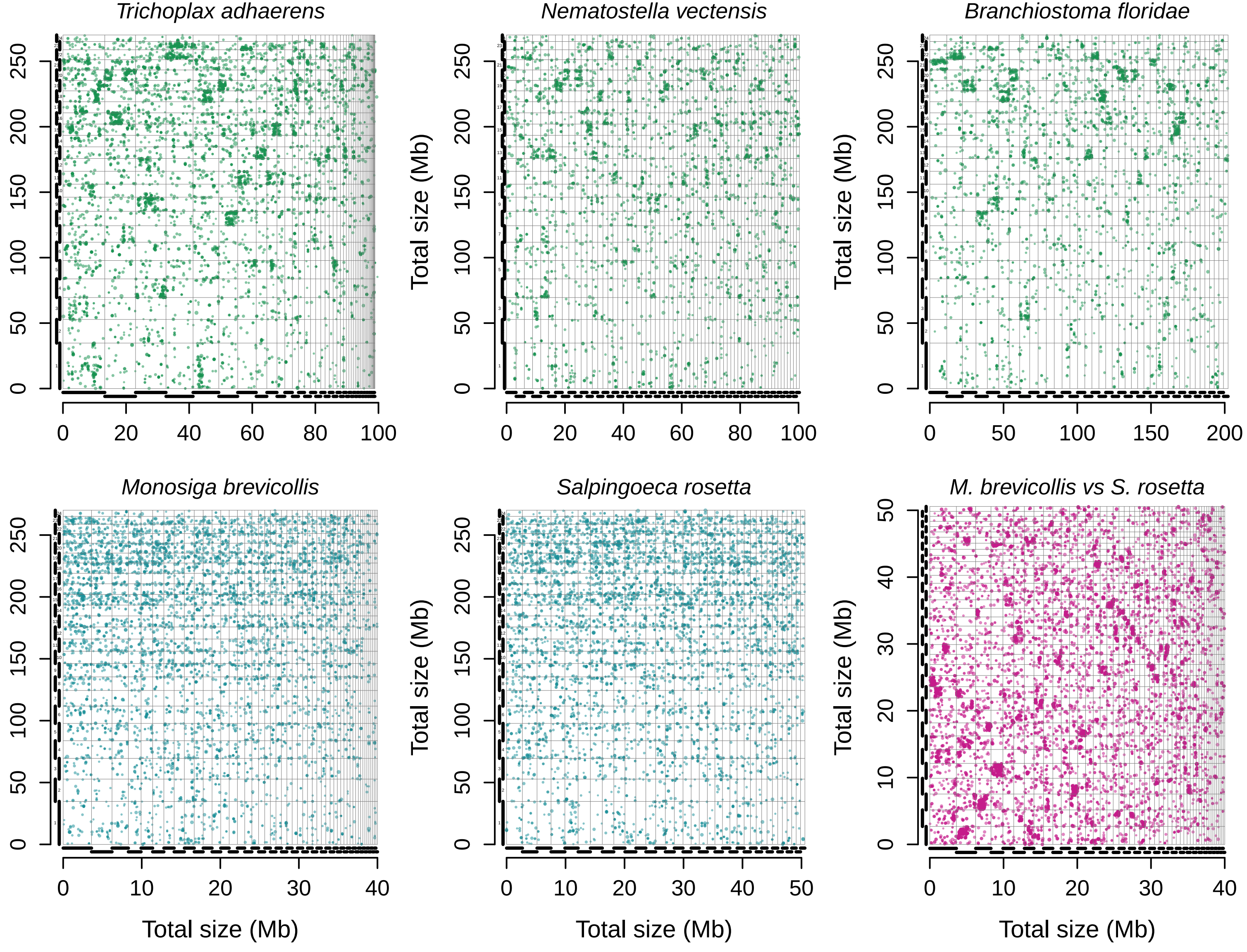

Trichoplax adhaerens
Nematostella vectensis
Branchiostoma floridae
Monosiga brevicollis
Salpingoeca rosetta
M. brevicollis vs S. rosetta
Total size (Mb)
Total size (Mb)
Total size (Mb)
Total size (Mb)
Total size (Mb)
Total size (Mb)
0
50
100
150
200
250
0
20
40
60
80
100
0
50
100
150
200
0
10
20
30
40
50
