# Supplementary material for: Tracing animal genomic evolution with the chromosomal-level assembly of the freshwater sponge Ephydatia muelleri
Source: Nat Commun. 2020 Jul 27;11:3676. doi: 10.1038/s41467-020-17397-w (PMC7385117; doi:10.1038/s41467-020-17397-w)
Supplement: Supplementary file 11 — Supplementary Data 7 [file 41467_2020_17397_MOESM11_ESM.zip › Supplementary_Data_7_Clustering_analyses_full_lists/README.pdf]

Part 1 Phylogeny analysis of 38 species (including *Ephydatia muelleri)*

Initial protein data sets used in this section following the steps in Pett e*t. all*, 2019 and the *Ephydatia muelleri* Homologs cluster analysis - All_species.tar.gz

Part 2 *Ephydatia muelleri* Homologs cluster analysis for finding unique genes

Supplementary Tables of Data

(Each table includes the data summarized from output files from the web tools used in this study in the section "Output Results")

8A - All Unique clusters EPMU.xlsx (3 Tabs)
8B - 550_Porifera_unique_proteins.xlsx (1 Tab)
8C - 7023_Demospongiae_unique_Proteins (EPMU,TEWH,AMQU)_summary.xlsx (10 Tabs)
8D - Not_Demospongia_Porifera_unique_proteins (SYCI,OSCA).xlsx (1 Tab)
8E - EPMU_GO_terms_2196.csv

Supplementary Figures F1-3:

8F - Figures_EPMU_REVIGO_treemap_for_over_all_GO_terms_BP_1.pdf
8G - Figures_EPMU_REVIGO_treemap_for_over_all_GO_terms_CC_2.pdf
8H - Figures_EPMU_REVIGO_treemap_for_over_all_GO_terms_MF_3.pdf

Fasta files analysez in the section

FA1 - EPMU_Proteins_unique.txt.fasta
FA2 - Porifera_proteins_unique.txt.fasta
FA3 - EPMU_ALL_unique_Demo_only_gene_names.txt.fasta
FA4 - AMQU_ALL_unique_Demo_only_gene_names.txt.fasta
FA5 - TEWI_ALL_unique_Demo_only_gene_names.txt.fasta
FA6 - OSCA_SYCI_proteins_unique.txt.fasta

Output Results

(Each Output zip file includes the tables and figures downloaded and analyzed during this study from the web tools used, PANZER and_REVIGO zip files include the relevant R scripts)

O1 - PANNZER_and_REVIGO-for_EPMU_clusters.zip
O2 - OrthoVenn2-for_EPMU_clusters.zip
O3 - PANNZER_and_REVIGO-550_Porifera_unique_proteins.zip
O4 - OrthoVenn2-550_Porifera_unique_proteins.zip
O5 - PANNZER_and_REVIGO-for_7023_Demospongiae_unique_Proteins .zip
O6 - OrthoVenn2-for_7023_Demospongiae_unique_Proteins.zip
O7 - PANNZER_and_REVIGO-for-Not_Demospongia_Porifera_unique_proteins (SYCI,OSCA).zip
O8 - OrthoVenn2-for-Not_Demospongia_Porifera_unique_proteins (SYCI,OSCA).zip

Part 3 *Ephydatia muelleri* Clusters analysis for Positively Selected Genes Expansion

8I_Ephydatia muelleri duplications.xlsx

PS-20200114T194047Z-001.zip
