# Supplementary material for: Tracing animal genomic evolution with the chromosomal-level assembly of the freshwater sponge Ephydatia muelleri
Source: Nat Commun. 2020 Jul 27;11:3676. doi: 10.1038/s41467-020-17397-w (PMC7385117; doi:10.1038/s41467-020-17397-w)

REVIGO Gene Ontology treemap

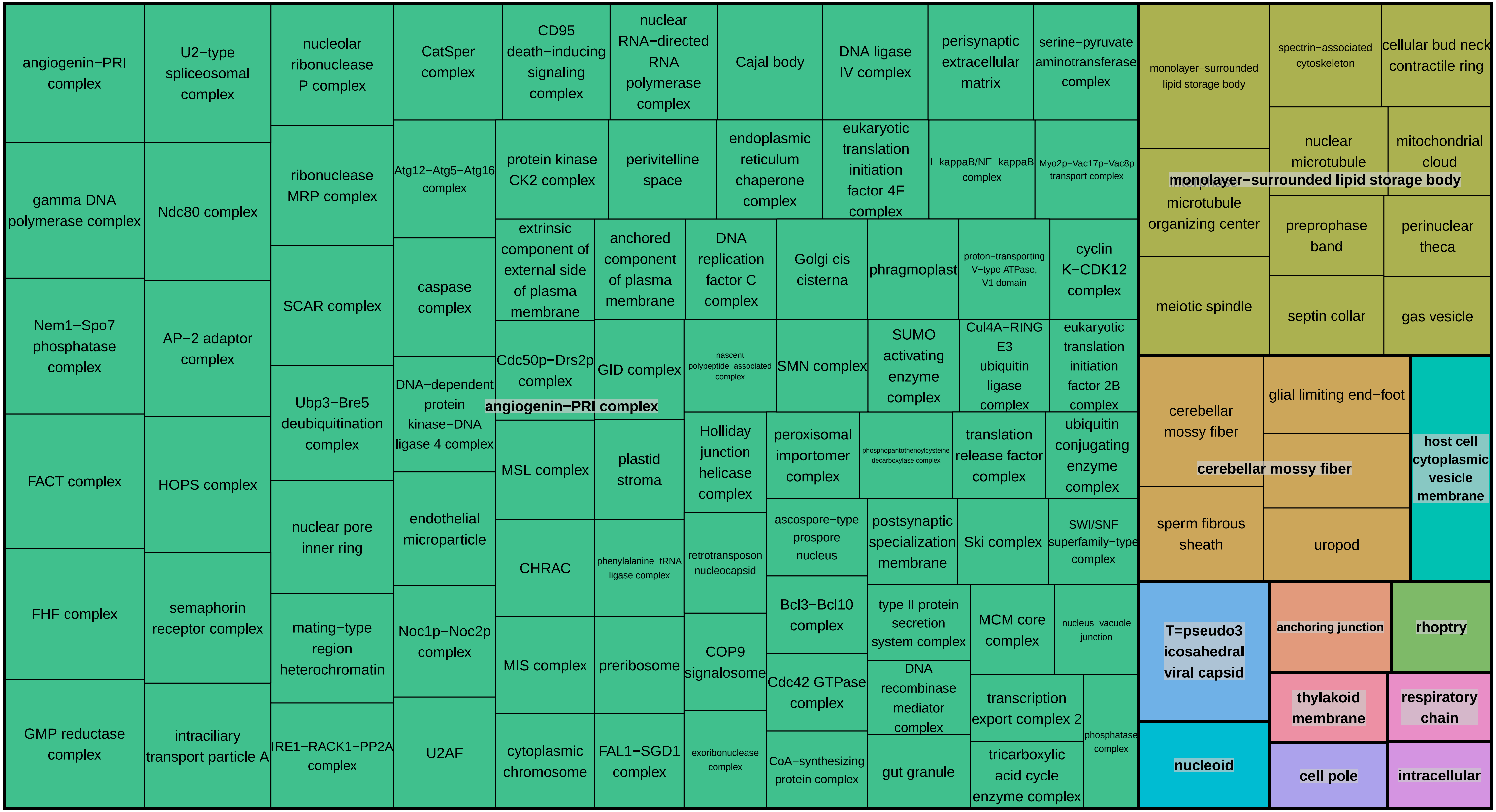

Supplement: Supplementary file 11 — Supplementary Data 7 [file Supp_Data_7G_Figures_EPMU_REVIGO_treemap_over_all_GO_terms_CC_2.pdf › Supplementary_Data_7_Clustering_analyses_full_lists/Supp_Data_7G_Figures_EPMU_REVIGO_treemap_over_all_GO_terms_CC_2.pdf]
