# Supplementary material for: Tracing animal genomic evolution with the chromosomal-level assembly of the freshwater sponge Ephydatia muelleri
Source: Nat Commun. 2020 Jul 27;11:3676. doi: 10.1038/s41467-020-17397-w (PMC7385117; doi:10.1038/s41467-020-17397-w)
Supplement: Supplementary file 11 — Supplementary Data 7 [file 41467_2020_17397_MOESM11_ESM.zip › Supplementary_Data_7_Clustering_analyses_full_lists/O1_PANNZER_and_REVIGO-for_EPMU_clusters.zip!/PANNZER_and_REVIGO/revigo-plot_CC_EPMu_Unique.pdf]

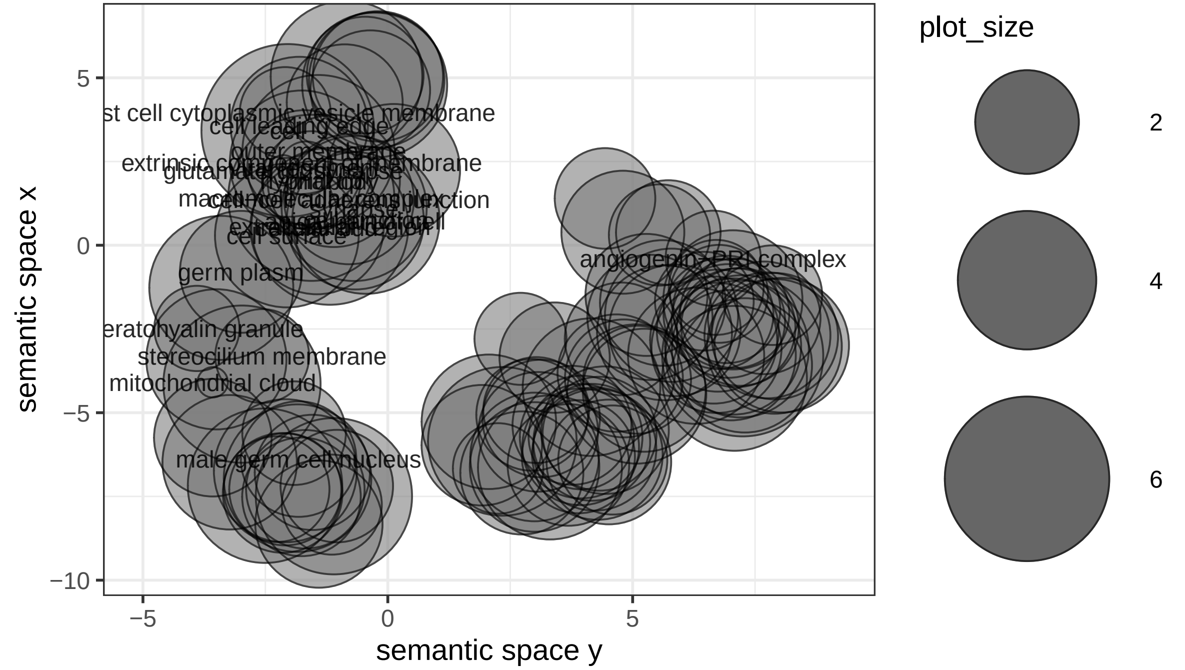

st cell cytoplasmic vesicle membrane
cell leading edge
outer membrane
cell surface
germ plasm
eratohyalin granule
stereocilium membrane
mitochondrial cloud
male germ cell nucleus
angiogenin-PRI complex
semantic space x
semantic space y
5
0
−5
−10
−5
0
5
plot_size
2
4
6
