# Supplementary material for: Tracing animal genomic evolution with the chromosomal-level assembly of the freshwater sponge Ephydatia muelleri
Source: Nat Commun. 2020 Jul 27;11:3676. doi: 10.1038/s41467-020-17397-w (PMC7385117; doi:10.1038/s41467-020-17397-w)
Supplement: Supplementary file 11 — Supplementary Data 7 [file 41467_2020_17397_MOESM11_ESM.zip › Supplementary_Data_7_Clustering_analyses_full_lists/O1_PANNZER_and_REVIGO-for_EPMU_clusters.zip!/PANNZER_and_REVIGO/revigo-plot_BP_EPMu_Unique.pdf]

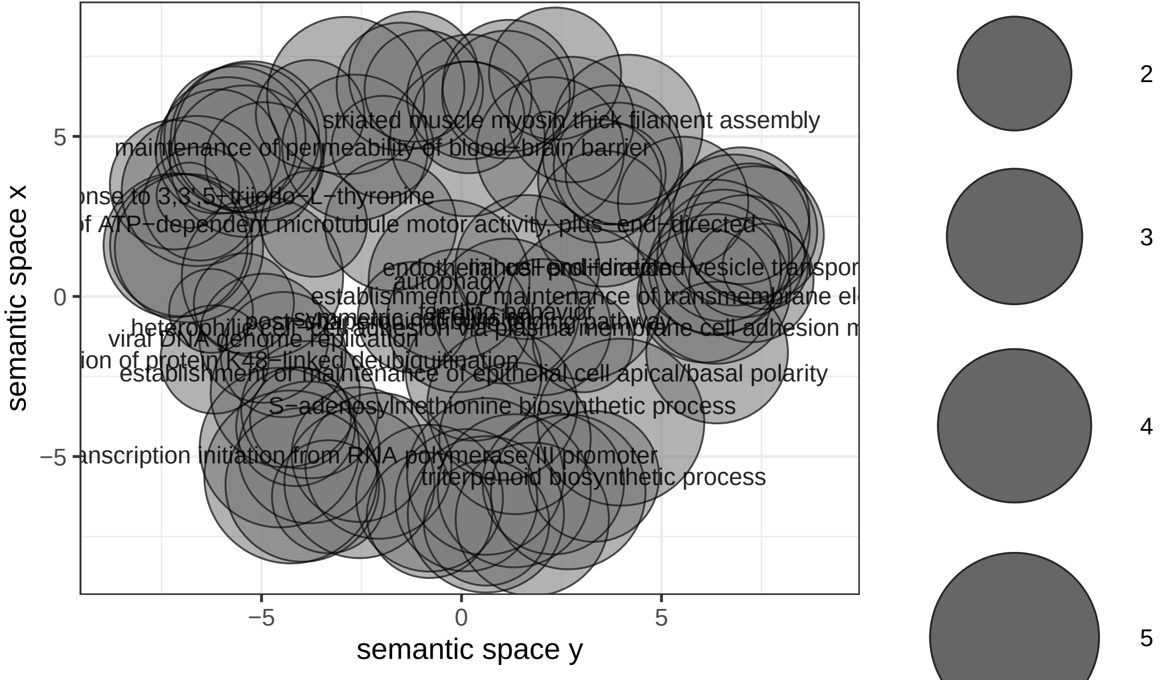

striated muscle myosin thick filament assembly
maintenance of permeability of blood−brain barrier
onse to 3,3',5−triiodo−L−thyronine
f ATP−dependent microtubule motor activity, plus−end−directed
autophagy
establishment or maintenance of transmembrane el
feeding behavior
heterophilic cell−cell adhesion via plasma membrane cell adhesion m
viral DNA genome replication
ion of protein K48−linked deubiquitination
establishment or maintenance of epithelial cell apical/basal polarity
S−adenosylmethionine biosynthetic process
anscription initiation from RNA polymerase III promoter
triterpenoid biosynthetic process
semantic space x
semantic space y
5
0
−5
−5
0
5
2
3
4
5
