# Supplementary material for: Tracing animal genomic evolution with the chromosomal-level assembly of the freshwater sponge Ephydatia muelleri
Source: Nat Commun. 2020 Jul 27;11:3676. doi: 10.1038/s41467-020-17397-w (PMC7385117; doi:10.1038/s41467-020-17397-w)
Supplement: Supplementary file 11 — Supplementary Data 7 [file 41467_2020_17397_MOESM11_ESM.zip › Supplementary_Data_7_Clustering_analyses_full_lists/O1_PANNZER_and_REVIGO-for_EPMU_clusters.zip!/PANNZER_and_REVIGO/revigo-plot_MF_EPMU_8147.pdf]

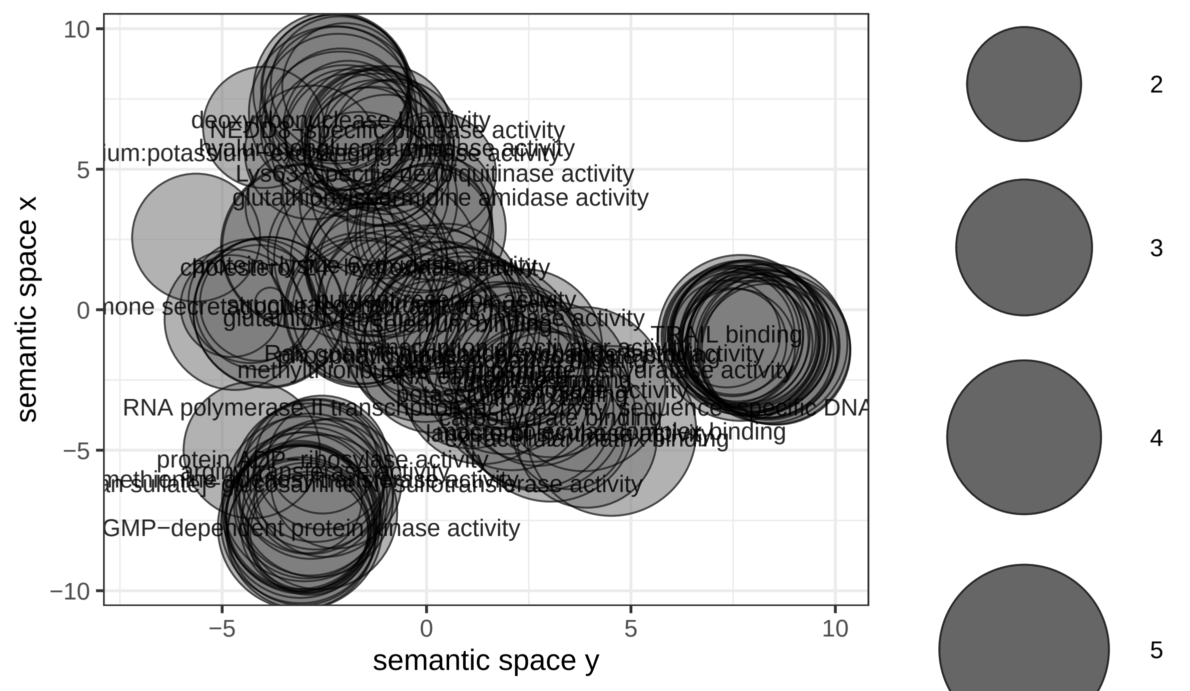

deoxyribonuclease II activity
NEDD8−specific protease activity
Lys63−specific deubiquitinase activity
glutathionylspermidine amidase activity
TRAIL binding
RNA polymerase II transcription factor activity, sequence−specific DNA
carbohydrate binding
macromolecular complex binding
extracellular matrix binding
protein ADP−ribosylase activity
arginyltransferase activity
GMP−dependent protein kinase activity
semantic space x
semantic space y
10
5
0
−5
−10
2
3
4
5
