# Supplementary material for: Tracing animal genomic evolution with the chromosomal-level assembly of the freshwater sponge Ephydatia muelleri
Source: Nat Commun. 2020 Jul 27;11:3676. doi: 10.1038/s41467-020-17397-w (PMC7385117; doi:10.1038/s41467-020-17397-w)
Supplement: Supplementary file 11 — Supplementary Data 7 [file 41467_2020_17397_MOESM11_ESM.zip › Supplementary_Data_7_Clustering_analyses_full_lists/O7_PANNZER_REVIGO-Not_Demospongia_Porifera_uniq(SYCI,OSCA).zip!/PANNZER_and_REVIGO/revigo-plot_CC_OSCA_SYCI.pdf]

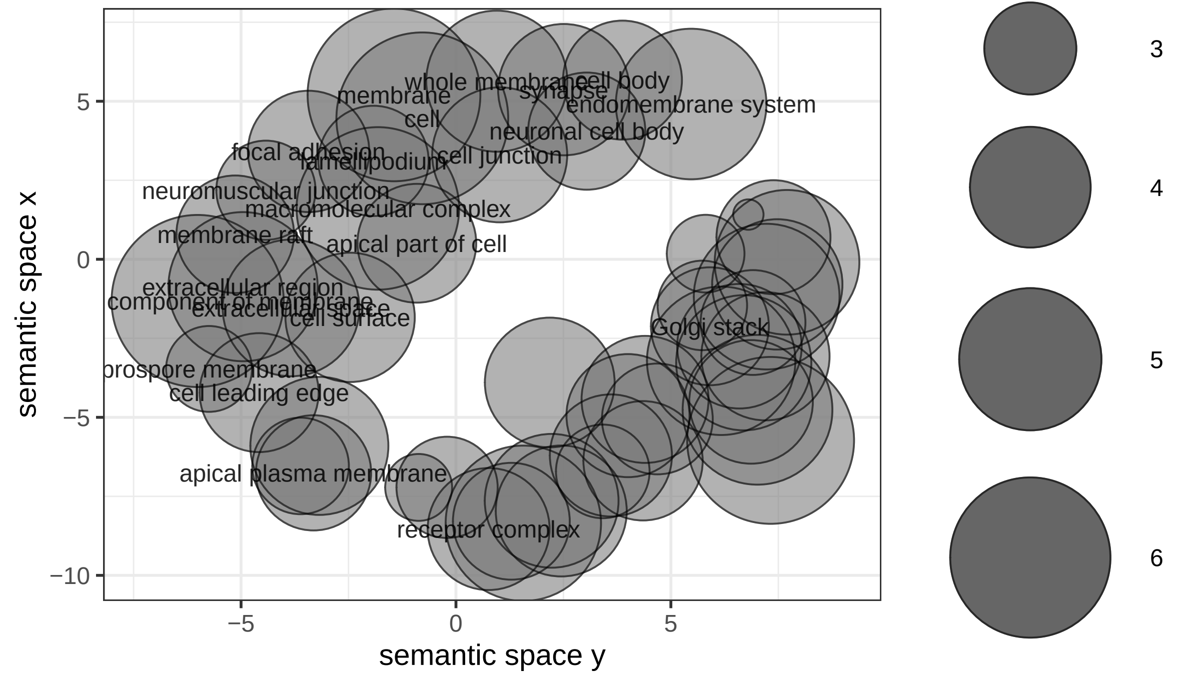
whole membrane
cell body
synapse
membrane
endomembrane system
cell
neuronal cell body
focal adhesion
lamellipodium
cell junction
neuromuscular junction
macromolecular complex
membrane raft
apical part of cell
extracellular region
component of membrane
extracellular space
cell surface
Golgi stack
prospore membrane
cell leading edge
apical plasma membrane
receptor complex
semantic space x
semantic space y
5
0
−5
−10
3
4
5
6
