# Supplementary material for: Tracing animal genomic evolution with the chromosomal-level assembly of the freshwater sponge Ephydatia muelleri
Source: Nat Commun. 2020 Jul 27;11:3676. doi: 10.1038/s41467-020-17397-w (PMC7385117; doi:10.1038/s41467-020-17397-w)
Supplement: Supplementary file 11 — Supplementary Data 7 [file 41467_2020_17397_MOESM11_ESM.zip › Supplementary_Data_7_Clustering_analyses_full_lists/O7_PANNZER_REVIGO-Not_Demospongia_Porifera_uniq(SYCI,OSCA).zip!/PANNZER_and_REVIGO/Warning_REVIGO.docx]

While parsing your data, warning(s) were encountered:

Go term 110148 was not found in the current version of the GeneOntology, dated 22:12:2016 16:59. GO term will be skipped.

Go term 140103 was not found in the current version of the GeneOntology, dated 22:12:2016 16:59. GO term will be skipped.

Go term 110111 was not found in the current version of the GeneOntology, dated 22:12:2016 16:59. GO term will be skipped.

Go term 99175 was not found in the current version of the GeneOntology, dated 22:12:2016 16:59. GO term will be skipped.

Go term 140102 was not found in the current version of the GeneOntology, dated 22:12:2016 16:59. GO term will be skipped.

Go term 62023 was not found in the current version of the GeneOntology, dated 22:12:2016 16:59. GO term will be skipped.

Go term 140096 was not found in the current version of the GeneOntology, dated 22:12:2016 16:59. GO term will be skipped.

Go term 140098 was not found in the current version of the GeneOntology, dated 22:12:2016 16:59. GO term will be skipped.

Go term 120035 was not found in the current version of the GeneOntology, dated 22:12:2016 16:59. GO term will be skipped.

Go term 120039 was not found in the current version of the GeneOntology, dated 22:12:2016 16:59. GO term will be skipped.

Go term 140097 was not found in the current version of the GeneOntology, dated 22:12:2016 16:59. GO term will be skipped.

Go term 140352 was not found in the current version of the GeneOntology, dated 22:12:2016 16:59. GO term will be skipped.

Go term 140056 was not found in the current version of the GeneOntology, dated 22:12:2016 16:59. GO term will be skipped.

Go term 120036 was not found in the current version of the GeneOntology, dated 22:12:2016 16:59. GO term will be skipped.

You have provided a huge list of GO terms, containing 533 terms in the BIOLOGICAL_PROCESS namespace, and only approx. 350 of these terms will be shown in the table below, or be available in the exported text table. REVIGO keeps the terms with the strongest p-values (or enrichements, depending what you had specified on the input form). Consider filtering the list by an external criterion (e.g. enrichment) before submitting to REVIGO.
