# Supplementary material for: Tracing animal genomic evolution with the chromosomal-level assembly of the freshwater sponge Ephydatia muelleri
Source: Nat Commun. 2020 Jul 27;11:3676. doi: 10.1038/s41467-020-17397-w (PMC7385117; doi:10.1038/s41467-020-17397-w)
Supplement: Supplementary file 11 — Supplementary Data 7 [file 41467_2020_17397_MOESM11_ESM.zip › Supplementary_Data_7_Clustering_analyses_full_lists/O7_PANNZER_REVIGO-Not_Demospongia_Porifera_uniq(SYCI,OSCA).zip!/PANNZER_and_REVIGO/revigo-plot_BP_OSCA_SYCI.pdf]

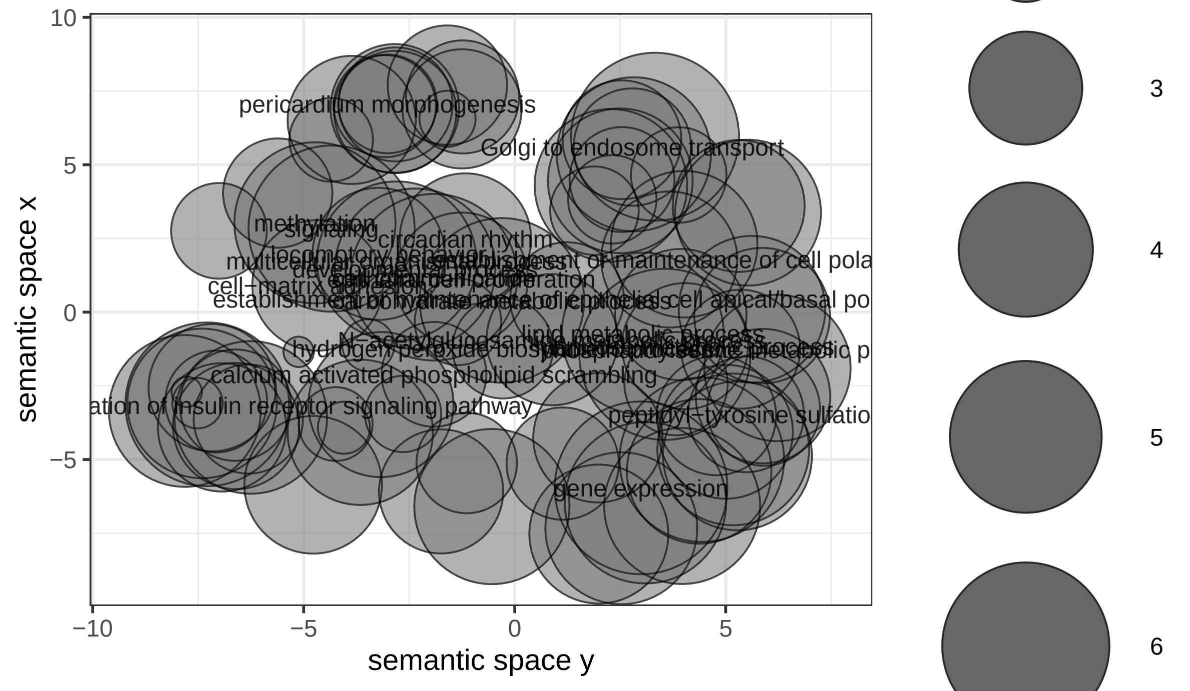

pericardium morphogenesis
Golgi to endosome transport
methylation
signaling
circadian rhythm
locomotory behavior
establishment or maintenance of cell pola
cell−matrix adhesion
carbohydrate metabolic process
lipid metabolic process
calcium activated phospholipid scrambling
ation of insulin receptor signaling pathway
peptidyl−tyrosine sulfatio
gene expression
semantic space x
semantic space y
10
5
0
−5
−10
3
4
5
6
