# Supplementary material for: Tracing animal genomic evolution with the chromosomal-level assembly of the freshwater sponge Ephydatia muelleri
Source: Nat Commun. 2020 Jul 27;11:3676. doi: 10.1038/s41467-020-17397-w (PMC7385117; doi:10.1038/s41467-020-17397-w)
Supplement: Supplementary file 11 — Supplementary Data 7 [file 41467_2020_17397_MOESM11_ESM.zip › Supplementary_Data_7_Clustering_analyses_full_lists/O3_PANNZER_and_REVIGO-550_Porifera_unique_proteins.zip!/PANNZER_and_REVIGO/revigo-plot_BP_197.pdf]

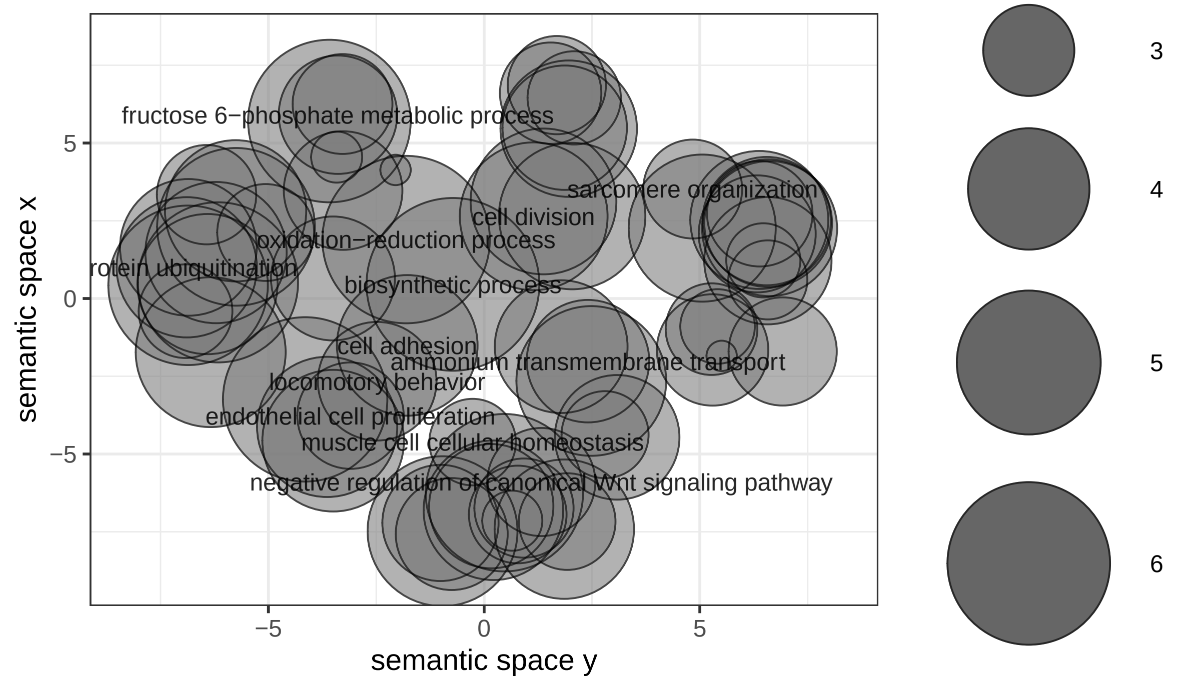

fructose 6−phosphate metabolic process
sarcomere organization
cell division
oxidation−reduction process
rotein ubiquitination
biosynthetic process
cell adhesion
ammonium transmembrane transport
locomotory behavior
endothelial cell proliferation
muscle cell cellular homeostasis
negative regulation of canonical Wnt signaling pathway
semantic space x
semantic space y
5
0
−5
−5
0
5
3
4
5
6
