# Supplementary material for: Tracing animal genomic evolution with the chromosomal-level assembly of the freshwater sponge Ephydatia muelleri
Source: Nat Commun. 2020 Jul 27;11:3676. doi: 10.1038/s41467-020-17397-w (PMC7385117; doi:10.1038/s41467-020-17397-w)
Supplement: Supplementary file 11 — Supplementary Data 7 [file 41467_2020_17397_MOESM11_ESM.zip › Supplementary_Data_7_Clustering_analyses_full_lists/O3_PANNZER_and_REVIGO-550_Porifera_unique_proteins.zip!/PANNZER_and_REVIGO/revigo-plot_MF_197.pdf]

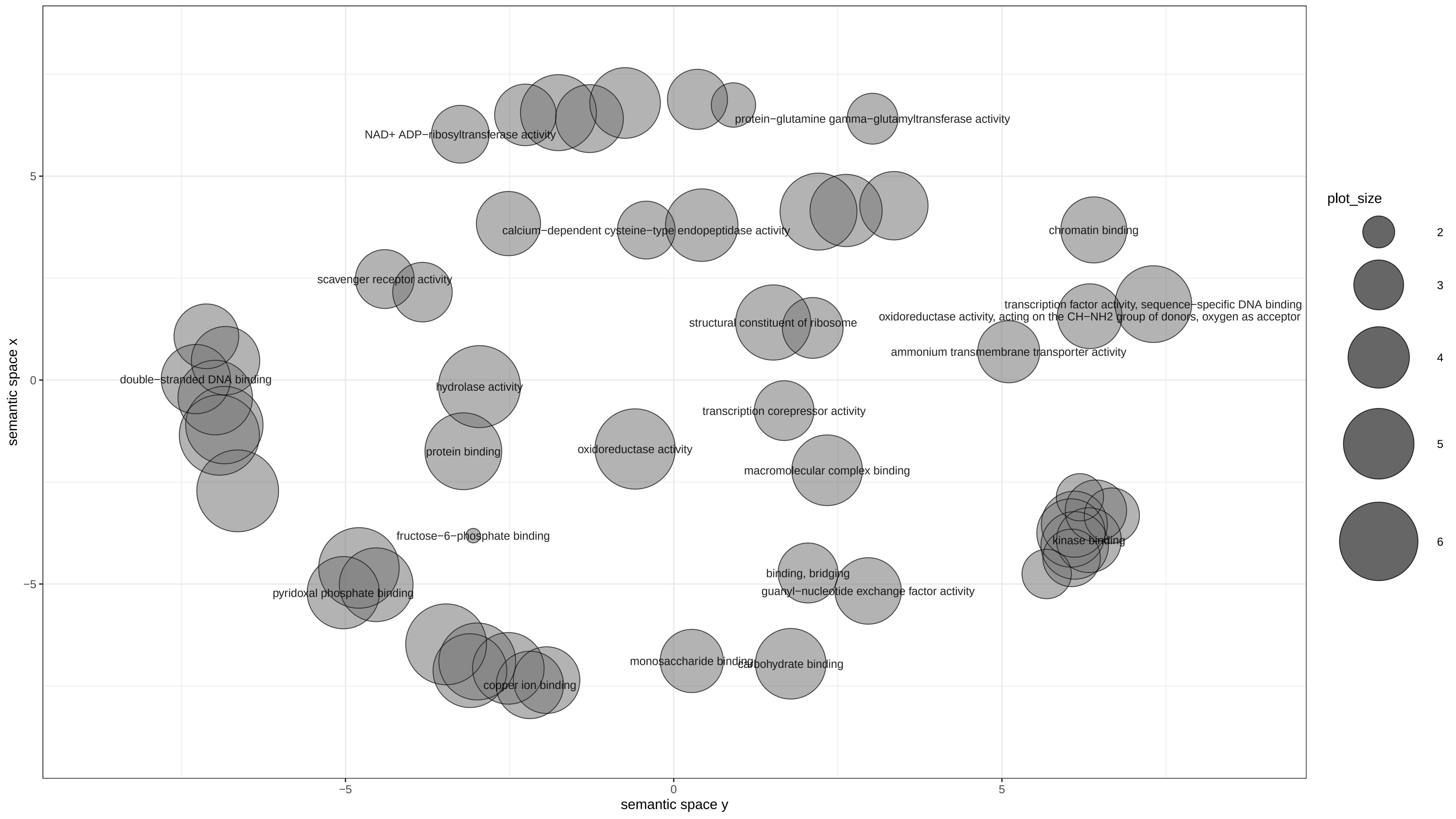

NAD+ ADP−ribosyltransferase activity
protein−glutamine gamma−glutamyltransferase activity
calcium−dependent cysteine−type endopeptidase activity
chromatin binding
scavenger receptor activity
transcription factor activity, sequence−specific DNA binding
oxidoreductase activity, acting on the CH−NH2 group of donors, oxygen as acceptor
structural constituent of ribosome
ammonium transmembrane transporter activity
double−stranded DNA binding
hydrolase activity
transcription corepressor activity
protein binding
oxidoreductase activity
macromolecular complex binding
fructose−6−phosphate binding
kinase binding
binding, bridging
guanyl−nucleotide exchange factor activity
pyridoxal phosphate binding
monosaccharide binding
carbohydrate binding
copper ion binding
semantic space x
semantic space y
5
0
−5
plot_size
2
3
4
5
6
