# Supplementary material for: Tracing animal genomic evolution with the chromosomal-level assembly of the freshwater sponge Ephydatia muelleri
Source: Nat Commun. 2020 Jul 27;11:3676. doi: 10.1038/s41467-020-17397-w (PMC7385117; doi:10.1038/s41467-020-17397-w)

REVIGO Gene Ontology treemap

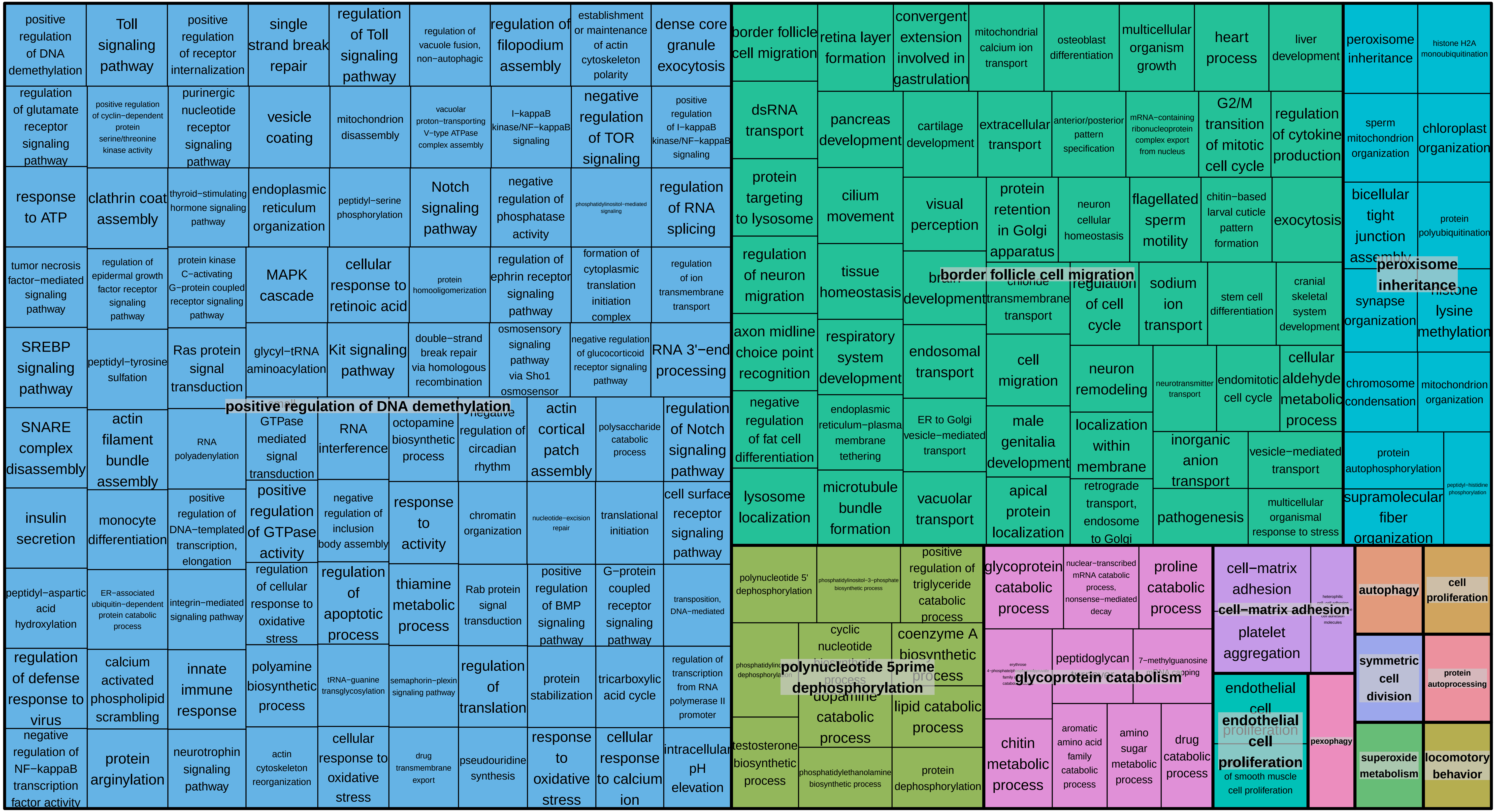

Supplement: Supplementary file 11 — Supplementary Data 7 [file 41467_2020_17397_MOESM11_ESM.zip › Supplementary_Data_7_Clustering_analyses_full_lists/O5_PANNZER_and_REVIGO-for_7023_Demospongiae_unique.zip!/PANNZER_and_REVIGO/revigo_treemap_Demo_unique_BP.pdf]
