# Supplementary material for: Tracing animal genomic evolution with the chromosomal-level assembly of the freshwater sponge Ephydatia muelleri
Source: Nat Commun. 2020 Jul 27;11:3676. doi: 10.1038/s41467-020-17397-w (PMC7385117; doi:10.1038/s41467-020-17397-w)
Supplement: Supplementary file 11 — Supplementary Data 7 [file 41467_2020_17397_MOESM11_ESM.zip › Supplementary_Data_7_Clustering_analyses_full_lists/O5_PANNZER_and_REVIGO-for_7023_Demospongiae_unique.zip!/PANNZER_and_REVIGO/revigo-plot_BP_Demo_unique.pdf]

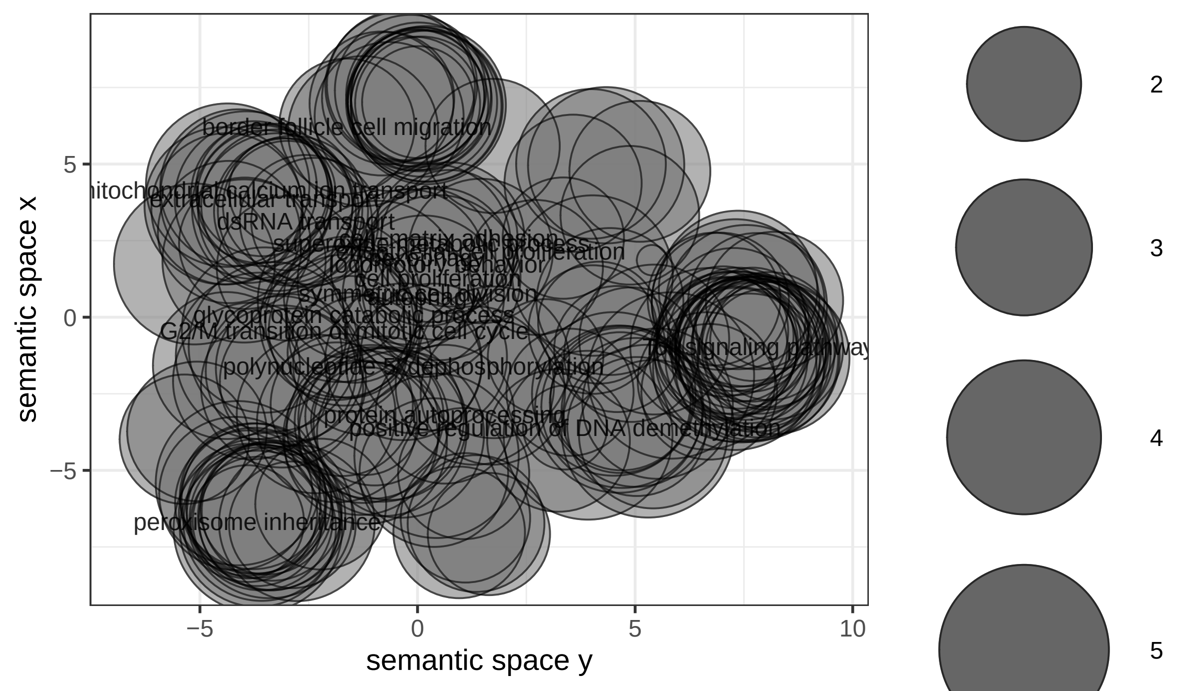

border follicle cell migration
extracellular transport
dsRNA transport
cell-matrix adhesion
locomotory behavior
cell proliferation
symmetric cell division
autophagy
glycoprotein catabolic process
G2/M transition of mitotic cell cycle
polynucleotide 5'-dephosphorylation
protein autoprocessing
positive regulation of DNA demethylation
peroxisome inheritance
semantic space x
semantic space y
5
0
−5
−5
0
5
10
2
3
4
5
