# Supplementary material for: Tracing animal genomic evolution with the chromosomal-level assembly of the freshwater sponge Ephydatia muelleri
Source: Nat Commun. 2020 Jul 27;11:3676. doi: 10.1038/s41467-020-17397-w (PMC7385117; doi:10.1038/s41467-020-17397-w)
Supplement: Supplementary file 11 — Supplementary Data 7 [file 41467_2020_17397_MOESM11_ESM.zip › Supplementary_Data_7_Clustering_analyses_full_lists/O5_PANNZER_and_REVIGO-for_7023_Demospongiae_unique.zip!/PANNZER_and_REVIGO/revigo-plot_Demo_unique_CC.pdf]

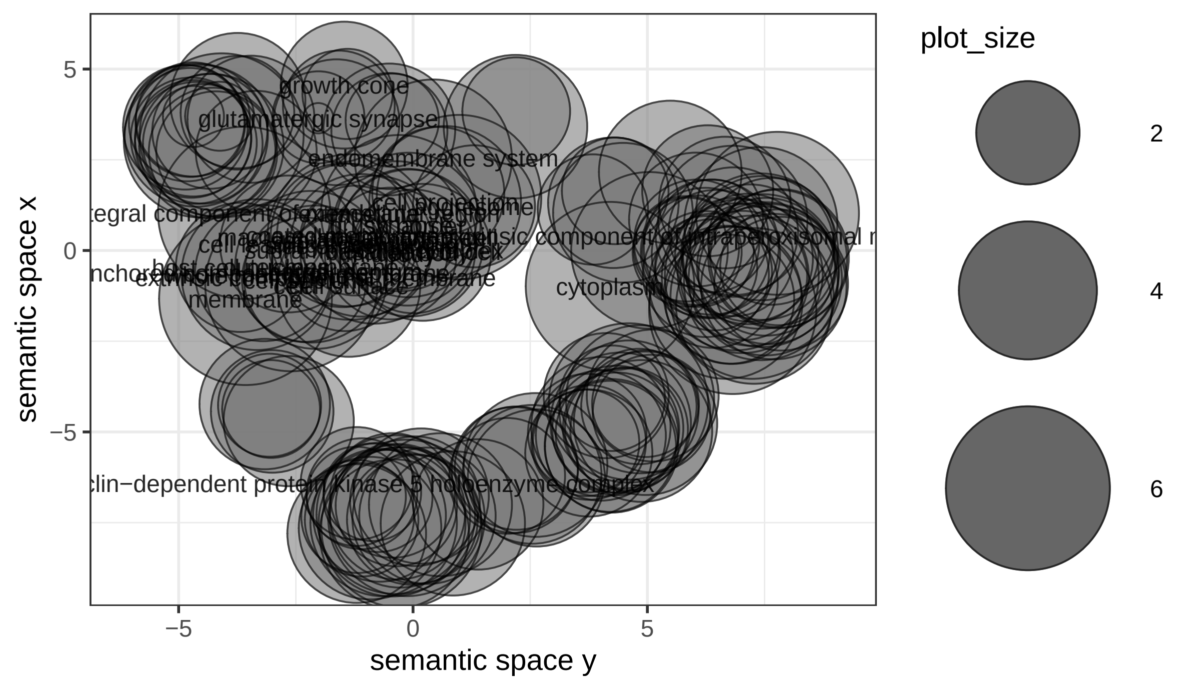

growth cone
glutamatergic synapse
endomembrane system
cell projection
cytoplasm
cell surface
membrane
clin−dependent protein kinase 5 holoenzyme complex
semantic space x
semantic space y
5
0
−5
−5
0
5
plot_size
2
4
6
