# Supplementary material for: Tracing animal genomic evolution with the chromosomal-level assembly of the freshwater sponge Ephydatia muelleri
Source: Nat Commun. 2020 Jul 27;11:3676. doi: 10.1038/s41467-020-17397-w (PMC7385117; doi:10.1038/s41467-020-17397-w)
Supplement: Supplementary file 138 [file 41467_2020_17397_MOESM11_ESM.zip › Supplementary_Data_7_Clustering_analyses_full_lists/README.odt]

Part 1 Phylogeny analysis of 38 species (including Ephydatia muelleri)

Initial protein data sets used in this section following the steps in Pett et. all, 2019 and the Ephydatia muelleri Homologs cluster analysis - All_species.tar.gz

Part 2 Ephydatia muelleri Homologs cluster analysis for finding unique genes

Supplementary Tables of Data

(Each table includes the data summarized from output files from the web tools used in this study in the section “Output Results”)

8A -  All Unique clusters EPMU.xlsx (3 Tabs)

8B -  550_Porifera_unique_proteins.xlsx (1 Tab)

8C - 7023_Demospongiae_unique_Proteins (EPMU,TEWH,AMQU)_summary.xlsx (10 Tabs)

8D - Not_Demospongia_Porifera_unique_proteins (SYCI,OSCA).xlsx (1 Tab)

8E - EPMU_GO_terms_2196.csv

Supplementary Figures F1-3: 

8F - Figures_EPMU_REVIGO_treemap_for_over_all_GO_terms_BP_1.pdf
8G - Figures_EPMU_REVIGO_treemap_for_over_all_GO_terms_CC_2.pdf
8H - Figures_EPMU_REVIGO_treemap_for_over_all_GO_terms_MF_3.pdf

Fasta files analysez in the section

FA1 - EPMU_Proteins_unique.txt.fasta

FA2 - Porifera_proteins_unique.txt.fasta

FA3 - EPMU_ALL_unique_Demo_only_gene_names.txt.fasta

FA4 - AMQU_ALL_unique_Demo_only_gene_names.txt.fasta

FA5 - TEWI_ALL_unique_Demo_only_gene_names.txt.fasta

FA6 -  OSCA_SYCI_proteins_unique.txt.fasta

Output Results

(Each Output zip file includes the tables and figures downloaded and analyzed during this study from the web tools used, PANZER and_REVIGO zip files include the relevant R scripts)

O1 - PANNZER_and_REVIGO-for_EPMU_clusters.zip

O2 - OrthoVenn2-for_EPMU_clusters.zip

O3 - PANNZER_and_REVIGO-550_Porifera_unique_proteins.zip

O4 - OrthoVenn2-550_Porifera_unique_proteins.zip

O5 - PANNZER_and_REVIGO-for_7023_Demospongiae_unique_Proteins .zip

O6 - OrthoVenn2-for_7023_Demospongiae_unique_Proteins.zip

O7 - PANNZER_and_REVIGO-for-Not_Demospongia_Porifera_unique_proteins (SYCI,OSCA).zip

O8 - OrthoVenn2-for-Not_Demospongia_Porifera_unique_proteins (SYCI,OSCA).zip

Part 3 Ephydatia muelleri Clusters analysis for Positively Selected Genes Expansion

8I_Ephydatia muelleri duplications.xlsx

PS-20200114T194047Z-001.zip
